# Transcriptomic signatures shaped by cell proportions shed light on comparative developmental biology

**DOI:** 10.1186/s13059-017-1157-7

**Published:** 2017-02-15

**Authors:** Sophie Pantalacci, Laurent Guéguen, Coraline Petit, Anne Lambert, Renata Peterkovà, Marie Sémon

**Affiliations:** 1 UnivLyon, ENS de Lyon, Univ Claude Bernard, CNRS UMR 5239, INSERM U1210, Laboratoire de Biologie et Modélisation de la Cellule, 15 parvis Descartes, F-69007 Lyon, France; 2 Laboratoire de Biométrie et Biologie Évolutive (LBBE), Université de Lyon, Université Lyon 1, CNRS, Villeurbanne, France; 3 Department of Teratology, Institute of Experimental Medicine, Academy of Sciences AS CR, Videnska 1083, 142 20 Prague, Czech Republic

**Keywords:** Comparative transcriptomics, Developmental biology, Transcriptomic signature, Temporal dynamics of gene expression, Heterochrony, Serial homology, Tooth

## Abstract

**Background:**

Comparative transcriptomics can answer many questions in developmental and evolutionary developmental biology. Most transcriptomic studies start by showing global patterns of variation in transcriptomes that differ between species or organs through developmental time. However, little is known about the kinds of expression differences that shape these patterns.

**Results:**

We compared transcriptomes during the development of two morphologically distinct serial organs, the upper and lower first molars of the mouse. We found that these two types of teeth largely share the same gene expression dynamics but that three major transcriptomic signatures distinguish them, all of which are shaped by differences in the relative abundance of different cell types. First, lower/upper molar differences are maintained throughout morphogenesis and stem from differences in the relative abundance of mesenchyme and from constant differences in gene expression within tissues. Second, there are clear time-shift differences in the transcriptomes of the two molars related to cusp tissue abundance. Third, the transcriptomes differ most during early-mid crown morphogenesis, corresponding to exaggerated morphogenetic processes in the upper molar involving fewer mitotic cells but more migrating cells. From these findings, we formulate hypotheses about the mechanisms enabling the two molars to reach different phenotypes. We also successfully applied our approach to forelimb and hindlimb development.

**Conclusions:**

Gene expression in a complex tissue reflects not only transcriptional regulation but also abundance of different cell types. This knowledge provides valuable insights into the cellular processes underpinning differences in organ development. Our approach should be applicable to most comparative developmental contexts.

**Electronic supplementary material:**

The online version of this article (doi:10.1186/s13059-017-1157-7) contains supplementary material, which is available to authorized users.

## Background

In multicellular organisms, organ development is a dynamic process manifested by changes in gene expression together with changes in tissue organization. Both interact to finally shape the adult phenotype. Therefore, studying the dynamics of gene expression during development helps to decipher developmental processes and their evolution across species [1, 2]. Historically, this was long done by looking at the expression of individual genes across conditions (time and/or mutants, organs, species). The raise of transcriptomics triggered a growing interest for identifying groups of genes with concerted expression differences across conditions. Clustering analysis of microarray or RNA-sequencing (RNA-seq) datasets are grounding numerous developmental studies and have provided helpful insights in comparative developmental biology (e.g. [3]). More recently, data mining with purposely developed tools allowed extracting information carried collectively by hundreds of genes, sometimes called “transcriptomic signatures,” in other fields such as toxicology and cancerology. These more global comparisons of transcriptomes have proven extremely useful to decipher cell lineages (e.g. [4, 5]), date embryonic samples (e.g. [6]), or extract organ or cell-specific genes during organogenesis and adulthood (e.g. [7]) (see also this review: [8]). In evo-devo, they were used as a measure of similarity, to assess the conservation of development depending on the stage and the phylogenetic scale (e.g. [9], hourglass pattern or inverted hourglass patterns in transcriptomes [10–13], developmental milestones in nematode species [13] or question homology relationships [14, 15] or the origin of new cell types [16]. The reciprocal question, how transcriptomic differences inform us of the developmental basis of morphological disparities between species or homologous organs, has been overlooked. Do transcriptomic signatures distinguish the development of homologous organs with different morphologies? Does the study of these signatures bring out an integrated view on differences in their developmental processes?

These questions can be more easily tackled, in a single species, in the context of serial homology. Serial organs are organs of the same “type,” but with different phenotypes, iterated at different positions of the same individual (e.g. leaves in plants or body appendages such as limbs in animals). Even when these organs are unambiguously of the same type, their shape can be quite different depending on their position (e.g. forelimb/hindlimb). Because they develop using the same genome, it is obvious that differential expression (DE) is the only factor promoting phenotypic differences, by building on context-dependent differences related to the position of the organ in the body. The comparison of gene expression in serial organs therefore helps to understand which differences in developmental processes result in organs that are similar, yet different.

In animals, the most commonly studied serial organs are body appendages, typically arthropod appendages and vertebrate limbs [17–20]. Molars, the model system used in this study, are another example, whether considered in the same or in opposite jaws [21, 22]. Research on serial appendages has put a strong focus on the role of what we called herein “identity genes.” These are genes with position-dependent expression, found specifically or in a specific combination in one type of appendage ([18–20]. The focus on these genes is consistent with the spectacular “homeotic transformations” of one appendage into another that arise when manipulating their function. These identity genes are most often transcription factors with homeodomains expressed specifically at organ initiation (e.g. homeotic genes for insect appendages and homeodomain transcription factors Tbx4 and Pitx1 for vertebrate limbs [19, 23], Dlx5,6 for jaws, and by extension, molars [22, 24]). They introduce specific regulations in an otherwise “default” developmental program made of the cascade regulation of thousands of common genes. These differential regulations are thought to be responsible for shape differences between serial appendages, yet we know little about them.

Indeed, these models focus on the initiation of development, somewhat discounting the rest of the process. In contrast, from the roots of evolutionary developmental biology, studies were interested in comparing the developmental sequence producing two serial organs (within and between species). They have shown numerous heterochronies, that is, temporal shifts in and/or between developmental processes (followed with marker genes or histological staining), trying to relate them to the final phenotypic outcome [25, 26].

A relatively poorly explored yet crucial question is to what extent, and how, gene expression differs during the development of serial appendages and how this reflects differences in developmental programs underpinning ultimate phenotypic differences. Previous studies comparing gene expression in serial organs lacked a detailed temporal resolution - when not focusing on a single stage (e.g. limbs [27], different type of teeth [28]). Moreover, they did not look for transcriptomic signatures, because they interpreted DE analysis at the level of the gene or group of genes. Here, we go beyond these limits by studying the dynamics of gene expression during the development of two phenotypically well-differentiated serial appendages, upper and lower molars in mouse. Below, we provide general background on molar development and more specific background on developmental differences between lower and upper molar.

**Fig. 1:**
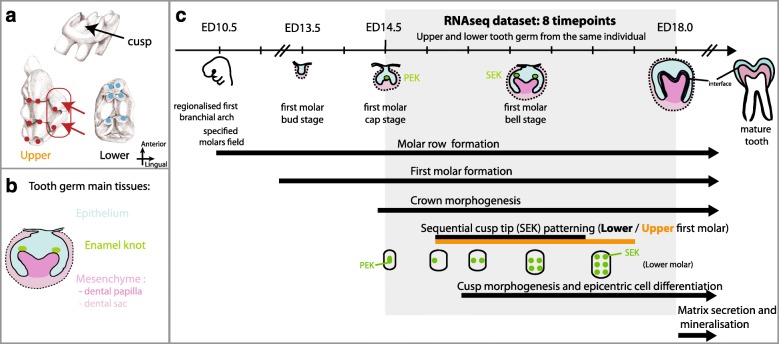
Mouse lower and upper first molars and their development. **a** *Top*: Lateral view of the mouse upper first molar showing its cusps. *Bottom*: Crown views of mouse lower and upper first molars. Drawings after [99]. **b** Simplified view of the tooth germ, composed of three main tissues: mesenchyme, epithelium, and (epithelial) enamel knot (EK), which serves as an epicenter for epithelium differentiation. **c** Scheme summarizing the main steps of molar development. At ED10.5, the lower and upper molars presumptive fields are specified in regionalized first branchial arch. At this point, the development of the molar rows is starting. By ED13.5, the first molars reach the bud stage: the oral epithelium has invaginated and tops a condensing mesenchyme of neural crest origin. Epithelium–mesenchyme interactions then lead to the formation of a signaling center in the invaginated oral epithelium, called the primary enamel knot (PEK), whose origin can be complex [100, 101]. The PEK directs the growth of dental epithelium around the further condensing and rapidly growing dental mesenchyme (bud-cap transition), resulting in the cap stage. This marks the beginning of the morphogenesis of the molar crown. Next, secondary enamel knots (SEK) are patterned sequentially. They sit at the tip of the future cusps and drive their morphogenesis, resulting in the bell stage. SEK patterning in lower molars is schematized with a domino and the period of SEK patterning in lower (*black bar*) versus upper molars (*orange bar*) is shown. SEK also act as epicenters for the progression of proliferation arrest and differentiation. Epithelial and mesenchymal cells situated at the inner interface between these two compartments differentiate into ameloblasts and odontoblasts, respectively, and at the very end of fetal life, those cells will start producing enamel and dentin, respectively. Note that the successive steps start sequentially but largely overlap because the downgrowth of the epithelium continues while cusps are patterned and the differentiation of the first cusps starts before the last cusps are formed

Using this model, we benefit from the strong body of work on molar evolution and development [29–31]. Although mouse lower and upper molars are both immediately recognizable as bona fide molars, exhibiting a mineralized crown with little hills named “cusps,” their shape is significantly different (Fig. 1). It is especially noticeable that the upper molar exhibits a supplementary row of cusps on its lingual side (red arrow on Fig. 1), on top of other differences (inclination, shape and arrangement of cusps, presence of accessory cusps). Present-day mouse lower and upper molar developmental programs have been shaped by at least 150 million years of evolution: molars evolved in early mammals from less complex teeth and since then mammalian lower and upper molar most often have had different morphologies [21]. Molars develop as jaw appendages and lower/upper molar specification is intimately linked to lower and upper jaw regionalization from the first branchial arch of the embryo (e.g. dependent on Dlx genes [22, 24, 31–33]). Of course, there are common principles for the development of lower and upper molars. The earliest steps of molar development (see Fig. 1 for more details) involve molar specific genes (e.g. as compared to incisor), like Barx1. These steps give rise to a molar tooth germ arranged in two compartments, epithelium and neural-crest derived ectomesenchyme (herein called mesenchyme for simplicity). Crown morphogenesis rests on shaping the epithelium–mesenchyme interface and differentiating enamel and dentin-secreting cells. These cells secrete a mineralizing matrix, which fixes the shape adopted by the soft tissue into a crown. This process depends heavily on self-organizing principles, involving epithelium–mesenchyme interactions through diffusible molecules and tissue mechanical properties [34, 35]. Key to the process are epithelial signaling centers called enamel knots (EK), which organize crown morphogenesis in time and space [36, 37]. The primary enamel knot (PEK) directs the folding of the epithelium around the condensing mesenchyme, defining the molar germ. Later, the secondary enamel knots (SEK) direct further folding of the epithelium, defining the cusps. These SEK are patterned sequentially (from embryonic day (ED)16.0) while molar germ grows [36, 37]. They also act as epicenters for the progression of proliferation arrest and differentiation.

Beyond these common principles, lower and upper molar developments, of course, show developmental and genetic differences. Our knowledge of these differences is, however, patchy, as a strong focus has been put on lower molar development ([29, 30], see also the database for expression in tooth: http://bite-it.helsinki.fi/ and transcriptomic data generated with microarray technology [38, 39]).

One such difference is the degree of participation of a vestigial bud to the anterior part of the first molar germ. Its relevance to final phenotypic differences between lower and upper molar is unclear [40]. A second difference is the persistence of lingual extension in the upper molar germ at ED16.5 [41], which paves the way for the formation of the third cusp row starting at ED17.5. Although it has not been put in these terms, this could be interpreted as a heterochrony, whereby a process shared by the two molars persists longer in the upper germ and is responsible for the supplementary cusp row.

Developmental genetics revealed a few genes specifically expressed and necessary in one type of molar and these are all lower-molar specific genes (Dlx5 and Dlx6 [22, 24], Nkx2.3 [28, 42], mesenchymal expression of Pitx1 [43]). On top of that, a number of genes expressed in both types of molars show differential requirements (Activin betaA [44], Bmp4 [45], Runx2 [46], Follistatin [47], Dlx1-2 [48, 49]). In some extreme cases, tooth development is arrested early in the mutant condition for one type of molar, but proceeds normally for the other type (e.g. Activin betaA, Bmp4, see references above). This indicates that the gene networks, although shared, can adopt very different states during lower and upper molar development. In line with this idea, a microarray study revealed numerous differentially expressed genes, including genes of key developmental signaling pathways and homeobox transcription factors [28]. However, this study was based on a single, and quite early, time point of the molar morphogenesis (ED14.5).

In this context, we took an original bioinformatic approach to compare the transcriptomic dynamics of the lower and upper molar germs, during the crown morphogenesis period (Fig. 1), that is, the period when lower and upper molar form, sequentially, their 6 and 8 cusps, respectively. In order to match gene expression dynamics with cusp formation, we not only sampled closely spaced RNA-seq transcriptomes (eight stages) but we also followed cusp formation using a cusp marker whose expression was evidenced in situ in the tooth germ.

Our in-depth study of transcriptomic signatures provides evidence of upper/lower specific differences in widely shared developmental mechanisms, and this, at three different levels. (1) First, the two developing teeth have a clear lower/upper transcriptomic identity carried by a large number of genes and maintained throughout molar development. This signature most notably stemmed from upper/lower biases in mesenchymal gene expression and relative abundance of the tooth mesenchymal component. This was consistent with experimental observations showing that the nature of mesenchyme and its abundance determine tooth final morphology. (2) Second, we evidenced a transcriptomic signature of heterochrony between the two teeth and related it to differences in the proportion of the territory occupied by a particular tissue (later forming molar crown cusps). Given our knowledge of molar development, this suggested specific differences in the activation-inhibition mechanisms ruling cusp formation in the two teeth. (3) Third, we evidenced a transient exaggeration of expression profiles in upper molar for genes involved in mitosis, adhesion, and migration. This counters the assumption that differences should accumulate during development and suggested transiently exaggerated morphogenetic processes. Taken together, this integrated transcriptomic analysis enabled us to formulate specific hypotheses of how the upper molar crown develops additional crown cusps. More generally, we note that all three transcriptomic signatures were shaped by differences in relative abundance of cell types within samples (i.e. proportion of mesenchymal/epithelial tissue, proportion of cusp tissue, proportion of mitotic or migrating cells), rather than to direct differences in gene regulation.

## Results

In this study, we targeted the period of lower and upper molar development during which most of the crown morphogenesis, and notably cusp patterning, happen (Fig. 1). We sampled lower and upper first molar tooth germs in eight individuals corresponding to eight consecutive stages, each separated by 12 h.

### Whole organ developmental transcriptomes carry an embedded signal of developmental timing

**Fig. 2:**
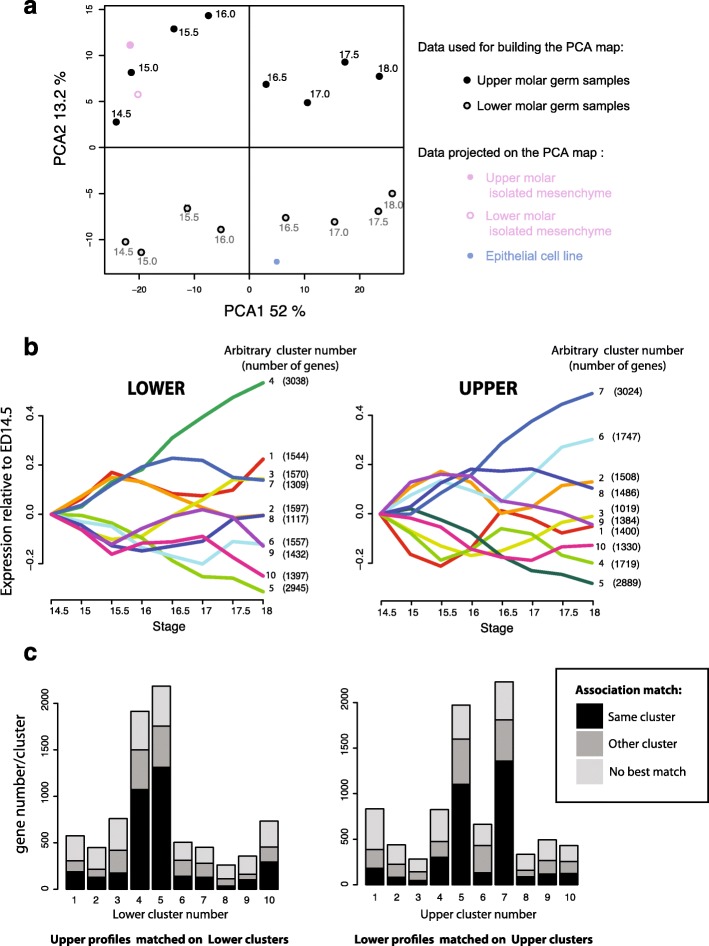
Lower and upper first molar germs share temporal dynamics of gene expression. **a** *Map* of 16 transcriptomes from upper (*black*) and lower (*gray*) molar germs, at eight stages of development, on the two first principal components of a principal component analysis (PCA). PCA1 orders samples with time, hence represents shared temporal variation. PCA2 separates upper and lower molar germs. The percentage of variation explained by each axis is indicated. Lower and upper first molar pure-mesenchyme transcriptomes (*pink*) and epithelial cell transcriptomes (*blue*) projected on this map are segregating near upper and lower samples, respectively. **b** Ten main temporal profiles of expression determined independently for the lower (left) and upper (right) tooth. Ten clusters were obtained by K-mean clustering (see “Methods”) and associated number of genes are indicated. Cluster numbering and color are arbitrary and do not indicate a correspondence between lower and upper clusters. Each profile represents the median of relative expression level (normalized with expression level at ED 14.5) for all genes associated to a particular cluster. **c** *Similarity of expression dynamics between upper and lower tooth*. *Left*: The height of each bar corresponds to the number of genes which lower time profile is robustly assigned to one of the ten main “lower” clusters (Pearson R > 0.7). In *black*, the number of genes whose time profile in upper molar associates best (and with R > 0.7) to the same lower cluster: such genes have similar time profile during lower and upper molar development. In *dark gray*, the number of genes for which expression profile in upper data associates best (and with R > 0.7) to another lower cluster. In *light gray*, the number of genes, for which the upper expression profile is not well associated to any lower profile (R < 0.7). *Right*: the experiment was done in the opposite direction, starting with genes whose upper time profile is robustly associated to a given upper cluster and challenging their lower time profile

As a starting point, we used multivariate analysis to extract the main axes of variation in gene expression within the time series for each type of tooth. We combined the lower molar samples, the upper molar samples, or all samples together. In each case, we found that the first axis represented about half of the total variation and ordered the samples according to developmental time (Fig. 2a, Additional file 1: Figure S1). This time ordering was extremely robust and resisted subsetting data, either by randomly sampling genes, by taking only the bottom (or top) 20% expressed sets of genes, or by retaining only tissue-specific genes (see Additional file 1: Figure S6). It is not caused by the fact that the expression of many genes is switched on and off between consecutive stages. We found a set of 641 genes showing such drastic pattern (i.e. very weakly expressed at one stage and expressed at least twice more in the consecutive stage). The removal of all genes does not impact the strength of principal component analysis 1 (PCA1) (61%), and the ordering of the samples.

### Most of the transcriptome series is similar between upper and lower molars

We next wanted to compare gene expression during lower and upper molar development. In a PCA combining lower and upper samples, the first axis of variation ordered samples with time (Fig. 2a). So, the main axis of variation was common to upper and lower molars, suggesting that the development path is largely common to these two types of tooth. This developmental time axis represented about 52% of variance, that is a bit less than 61–64% of variance (which was observed in lower or upper-specific PCAs, see Additional file 1: Figure S1). This is however high, suggesting that the vast majority of time variation in gene expression is shared by the lower and the upper developing molars.

This strong similarity was also shown by the analysis of the main clusters of developmental expression time series. We extracted ten main developmental profiles in upper and in lower tooth (see “Methods”). These profiles are shown in Fig. 2b, together with the number of genes that were assigned to each of them. Linearly increasing/decreasing profiles made up one-third of the genes transcribed in each organ (34%, clusters 4 and 5 in the lower tooth or clusters 7 and 5 in the upper tooth).

Some of the clusters (1, 2, 4, and 5 for the lower tooth and 5 and 7 for the upper tooth) were strongly enriched in genes involved in development, morphogenesis, signaling and adhesion, and cell proliferation and differentiation (Additional file 1: Figure S2) including genes that are annotated with especially relevant developmental processes, such as odontogenesis, skeletal system, or appendage development. Clusters 9 and 10 of the lower tooth (which have resembling profiles) and cluster 1 of the upper tooth were enriched in genes involved in mitosis (e.g. GO mitotic nuclear division; note that this enrichment is much stronger and the profile much sharper for the upper cluster). The profile of this upper cluster 1 resembled upper cluster 4, which was enriched in genes involved in DNA metabolism and DNA replication, and may thus be the counterpart for the G1/S phase of the cell cycle. Taken together these clusters suggested that lower and upper tooth germs are depleted in proliferating cells at ED 15.5, an effect that was sharper in the upper molar. Other clusters show no general pattern of functional enrichment, but nevertheless revealed small groups of genes involved in specific processes (e.g. N-glycosylation for lower and upper cluster 3; cell migration and integrin signaling pathway for upper cluster 6; synaptic transmission for upper cluster 9, lamellipodium organization for upper cluster 10; inflammatory response for upper cluster 2). Finally, some clusters were only poorly enriched for specific functions (e.g. clusters 6, 8 of the lower tooth).

We then used these clusters as a way to quantify the similarity between development in the upper and in the lower tooth. From the ten main representative profiles extracted from lower molar data, we retained only the genes which were robustly associated to one of these representative profile (see “Methods” for the test employed), that is, 8187 genes. Among these genes, we counted the number of genes which were associated together in the same profile in the upper and in the lower tooth. Such genes are expected to have more or less the same temporal expression profile in both organs. This was the case much more often than expected (Chi-squared test, *P* value <10-16), and represented altogether 56% of the genes. The same result was obtained with the reciprocal analysis (Fig. 2c, right), mapping expression from the upper tooth to the lower tooth.

### Intrinsic difference in transcriptome between upper and lower tooth

#### An intrinsic difference in transcriptome between upper and lower tooth is carried by a large number of genes

Although most of the gene expression was similar between the upper and the lower tooth, the differences were far from negligible. This was true in terms of time profiles, as exemplified by the frequency of genes that do not map to the same cluster in the upper and the lower tooth (44%, Fig. 2c) and by the importance of the second axis of the PCA. This second axis of variation segregated samples between the upper and lower molars, whatever the stage considered (Fig. 2a), and represented 13.2% of the total variation.

We first wondered whether this pattern could be caused by specific or strongly biased genes that would mark a clear lower versus upper molar identity, as identified for early jaws [50]. A single gene, Nkx2-3, was specifically expressed in the lower molar and we found no gene specific for the upper molar, although the top upper gene, Pou3f3, was about ten times more expressed in the upper molar. If fact, when taking all samples from each kind of tooth in the large-scale dataset as replicates (so in total, eight replicates for upper and eight for lower molar), we found 1347 genes (out of 14,808) differentially expressed (“upper/lower DE genes,” adjusted *P* value < 0.1), out of which only 83 show more than a twofold excess difference (see Additional file 2: Table S1). This included genes known for their role in molar or jaw specification (like Dlx5 and 6, Pou3f3, and two associated non-coding RNAs (ncRNA), 2900092D14Rik, 2610017I09Rik [50], Pitx1 [43]). We concluded that there were relatively few genes that were consistently biased with a fold change over 2 throughout stages. On a developmental point of view, however, these consistently biased genes were possibly sufficient to provide and sustain different orientations for upper and lower molar development.

Are those consistently biased genes sufficient to explain the genomic signature that separates the upper and the lower tooth? To answer this question, we looked how far this genomic signature would resist the removal of differentially expressed genes. Removing the 83 above-mentioned genes had a marginal effect on the second axis of PCA (11.4% of variation explained instead of 12.9% with the 83 genes). In fact, the second axis of the PCA still separated the upper and lower samples and represented a significant amount of the total variation, even when all differentially expressed genes were removed: after removing 1347 DE genes that were found when the eight stages are taken as replicates, the axis that splits upper and lower tooth represented 9.3% of the total variation. Upper/lower DE genes can also be estimated taking time into account (DESeq2, adjusted *P* value < 0.1), which is less stringent and resulted in 3155 DE genes: after removing these genes, the axis that splits the upper and the lower tooth represented 7.8% of the total variation. We concluded that the upper/lower transcriptomic signature was not only carried by sets of genes that are moderately to strongly biased throughout the developmental period, but also by more subtle gene expression differences in a very large number of genes.

#### Upper/lower transcriptomic differences are partly driven by differences in proportions between tooth epithelial and mesenchymal compartments

**Fig. 3:**
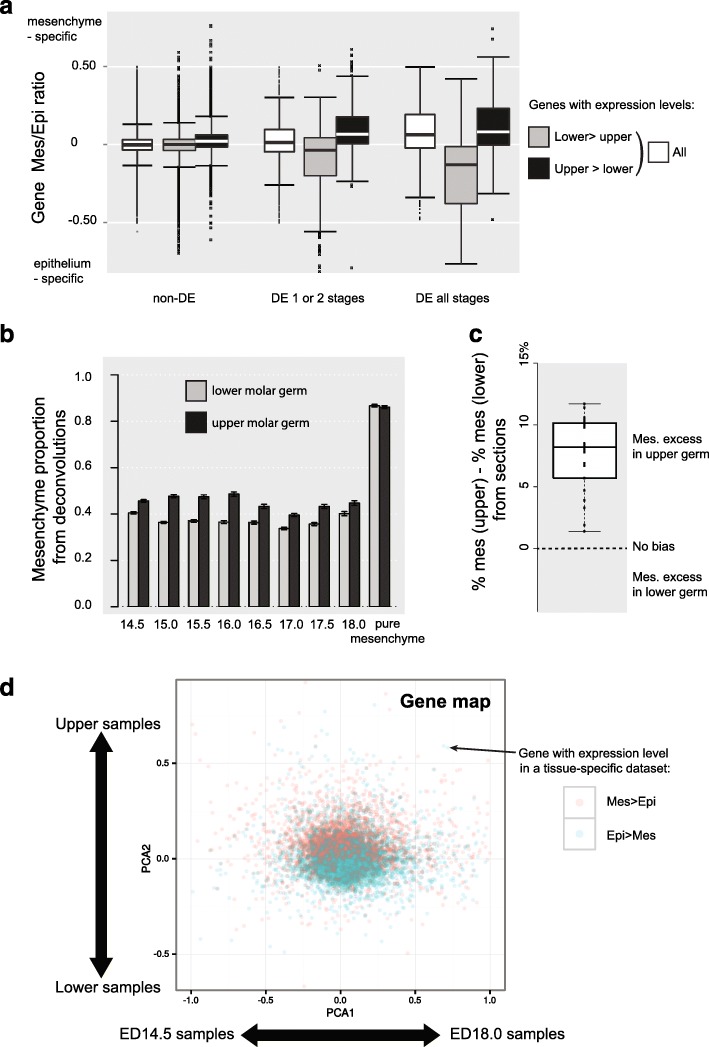
The upper molar germ is enriched in mesenchyme. **a** Boxplot showing the distribution of an index of mesenchyme specificity, for the totality of genes (*white*) and for genes more expressed in upper (*black*) or in lower (*gray*) molar germ. This index was measured as the ratio of expression levels between mesenchyme and epithelium (median of log ratio(mesenchyme/epithelium) at 12.0, 13.0, and 13.5 dpc, estimated using microarray data [39]. *non-DE* genes non-significantly biased between upper and lower tooth, All genes significantly biased for at least one stage, DE 1 or 2 stages genes significantly biased for one or two developmental stages, DE all stages genes significantly biased for all developmental stages. **b** Mesenchyme proportions estimated from deconvolutions for our eight developmental stages. Markers were obtained from bite-it database (11 for EK, 12 for epithelium, and 14 for mesenchyme). Confidence intervals were extracted by resampling (500 bootstraps). RNA-seq samples of mesenchyme isolated from lower and upper molar germs [45] were used as positive controls. **c** Boxplot showing the difference between mesenchyme proportion between 24 pairs of upper and lower molar germs, taken between ED 14.5 and ED 17.5. Mesenchyme proportion is expressed as a percentage of mesenchyme volume relative to total germ volume, as measured on complete series of histological sections. **d** Map showing the location of the genes on the two first principal components of a PCA analysis obtained from upper (*black*) and lower (*gray*) molar germs, at eight stages of development. The interpretation of PCA axes (developmental timing for PCA1 and upper/lower for PCA2, respectively) is reminded with arrows. Genes are colored according to their tissue-specificity in a tissue specific dataset ([39]; expressed mesenchyme > epithelium in *pink*; expressed epithelium > mesenchyme in *blue*)

Because experimental work has suggested that the mesenchyme carries molar identity at the examined stages [51], we wondered whether the above lower/upper DE genes would rather be mesenchyme-specific genes. We took advantage of a previous study that had sampled epithelium and mesenchyme transcriptomes separately at an earlier stage [39] to attribute a tissue specificity score, by simply calculating a mesenchyme/epithelium ratio. Then, we extracted stage-by-stage differentially expressed genes, by considering seven pairs of consecutive developmental stages as replicates (see “Methods”). We obtained 1146 genes that differed between upper and lower tooth for only a portion of the whole time series (one or two such “pairs of consecutive stages”), 1646 genes that differed at in all comparisons (seven such “pairs of consecutive stages”), and 10,933 genes that never differ significantly, in any pair comparison. We found that genes that differ between upper and lower molar were on average significantly biased towards mesenchyme specificity (Wilcoxon test, *P* value < 10e-16, Fig. 3a in white).

When we further separated these sets in two categories, depending whether they are enriched in lower or in upper molar (genes assigned to “lower” in gray or “upper” in black in Fig. 3a), we were, however, surprised that the tissue specificity of lower and upper genes was on average significantly different: upper genes are clearly more mesenchyme-specific than lower genes (Fig. 3a, Wilcoxon test *P* value < 10e-16 in all comparisons of mesenchyme ratio for the three gene sets). This was even more pronounced for genes that were biased at all stages, but interestingly, this was also true, although to a minor extent, for genes that were not significantly differentially expressed (Fig. 3a, *P* value < 10-16).

Such bias impacting a large part of the transcriptome, including non-DE genes, could be explained if the proportions of epithelium and mesenchyme differed between lower and upper molar, the lower molar being enriched in epithelium and the upper molar enriched in mesenchyme. We performed deconvolutions (see “Methods”) using three sets of marker genes (markers from bite-it [52], 11 genes for enamel knot –EK--, 12 for epithelium, and 14 for mesenchyme) to estimate the relative proportions of mesenchyme, epithelium, and enamel knot in the molar germs. We show that the estimated proportions of mesenchyme were systematically higher in upper teeth, especially in earlier stages (Fig. 3b, the difference was significant at each developmental stage, obtained by paired Wilcoxon tests on bootstrapped values, *P* values < 10-16). Similar results were obtained from another set of markers defined using microarray data (Additional file 1: Figure S3). Such a difference had not been pointed before, thus we performed a direct quantification of epithelium and mesenchyme compartments on histological sections taken throughout development and we found that the upper molar was indeed enriched in mesenchyme as compared with the lower molar throughout the time window of interest (Fig. 3c, Wilcoxon test, *P* value = 2*10-5; see also quantification on three-dimensional (3D) reconstructed tooth germs, Additional file 1: Figure S4). These results thus highlighted that the upper molar germ was enriched in mesenchyme relative to the lower molar germ throughout the period of cusp formation.

Next, we reasoned that such a pervasive effect on the transcriptome, seen even for non-DE genes, could typically shape the second axis of PCA (PCA2). This prompted us to examine the mesenchyme specificity of the genes contributing to this axis. One main advantage of PCA is that it is possible to extract coordinates for each gene. Genes with large coordinates contribute the most to the axis. We found a clear correlation between the coordinates in PCA2 and a mesenchyme/epithelium ratio extracted from published tissue-specific expression obtained at earlier stages using microarray data (Fig. 3d [39, 51], Pearson R = 0.280, *P* value <10-16). Thus, genes colocalizing with the upper molar tend to be mesenchyme-specific, while genes co-localizing with the lower molar tend to be epithelium-specific, as seen above. Finally, it was also possible to map external datasets on a PCA. In such mapping, pure tooth mesenchyme RNA-seq data (taken from upper and lower tooth from stage ED13.5 [45]) co-localized with early upper tooth samples when they were projected on the PCA plot, while epithelial cell lines (not even from tooth nor mouse: human mammary epithelial cells [53]) located on the side of lower tooth samples. All these data illustrated that PCA2 discriminates genes with epithelial and mesenchyme biases.

We then wished to evaluate how far differences in the epithelium–mesenchyme ratio take part in shaping expression differences between the lower and upper molars by two complementary approaches. First, we removed the genes whose expression is strongly biased in mesenchyme or in epithelium pure tissue transcriptomes (from [39]). We retained 9263 genes whose epithelium–mesenchyme ratio is less biased than the third quartile. Using this set of genes to draw a PCA still separated the upper and the lower tooth on the second axis, but the importance of this axis decreased (this second axis represented 10.8% of the total variance, which was slightly less than 13% on the total set of genes). Second, we simulated a reasonable increase of mesenchyme proportion in a lower ED 14.5 sample (see “Methods”) and mapped this artificial sample on the PCA. This artificial sample was shifted along the second axis; however, it was not sufficient to morph a lower tooth into an upper tooth: it covers only 23–35% of the upper/lower distance on axis 2 between lower and upper tooth germ at this stage. We concluded that pervasive differences in gene expression due to tissue proportion were clearly taking part in shaping axis PCA2, although they were not sufficient to explain it.

#### Upper/lower transcriptomic differences are also driven by differences within compartments

We then reasoned that pervasive differences in gene expression not due to tissue proportion, but constantly opposing lower and upper molars, should also be responsible for shaping this axis 2. To get rid of tissue proportion effects, we again made use of a previously published RNA-seq dataset of pure mesenchyme. This was obtained from laser dissected tissues, in a lower molar germ that is one day younger than the youngest sample of our dataset (ED13.5, that is, before cap stage [45]). As seen in the previous section, the upper and lower mesenchyme samples mapped correctly on the second axis of the PCA (Fig. 2a). They were more closely mapped than samples extracted from whole tooth germ, and their distance only corresponded to 22–41% of the upper/lower distance obtained on whole tooth germ samples (a more realistic estimate, if we consider only early stages of development, would be closer to 25% of the upper/lower distance). This confirmed that the transcriptomic differences shaping PCA2 are not only a matter of epithelium *versus* mesenchyme proportion, but also (at least) of mesenchyme quality. Moreover, this suggested that these differences are already established before cap stage, as the mapped transcriptomes were younger (13.5 days).

We have shown earlier that there was a set of constantly biased genes (1347 genes with upper/lower bias), most of them being only slightly to moderately biased. The data above suggested that, even if part of the upper/lower difference is driven by epithelium–mesenchyme proportions, another part is related to a different mesenchyme quality in upper molar compared to lower molar. To confirm this, we performed two additional experiments. When performing a new PCA after removing those 1347 genes from our dataset and mapping again the pure mesenchyme samples, the distance drops to 8% on average (median: 8.3%, range: 5.2–10.1%) of the upper/lower distance obtained on whole tooth germ samples. Reciprocally, performing the same experiment on a dataset consisting only of these 1347 genes, the distance between pure mesenchyme samples increases to 46% (median: 46%, range: 32.4–59.3%) of the upper/lower distance obtained on whole tooth germ samples. The sensitivity of these mapping experiments to this set of 1347 genes was a good indication that this set is partly shaped by constant upper-lower differences (at least) in the mesenchyme, already seen at ED13.5. Said differently, part of the lower/upper variation of gene expression making up this second axis was also found in these mesenchymal datasets. It could represent one-fourth to one-third of the transcriptome difference between upper and lower whole tooth germs and would therefore be attributable to differences in the level of expression, irrespective of differences in tissue compartment proportions.

In summary, our data suggest that lower and upper molar transcriptomes exhibit clear signatures of identity: “lowerness” versus “upperness.” This signature is partly made by differences in tissues proportions, the upper molar germ being enriched in mesenchyme as compared with the lower molar germ, but also by differences in expression within tissues (at least mesenchyme), which are established as early as before the cap stage (ED13.5).

### Early peak of the difference in transcriptome between upper and lower tooth

**Fig. 4:**
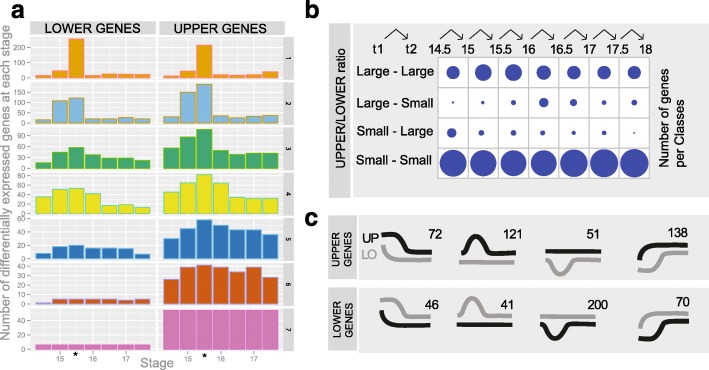
Time-dependent differences in upper/lower gene expression. **a** Number of differentially expressed genes for seven consecutive periods. We defined these seven periods by considering pairs of consecutive developmental stages as replicates (i.e. ED14.5–15.0, ED15.0–15.5, etc.). We extracted differentially expressed genes at each of these seven periods and split genes into two categories: those with a higher expression level in lower molar and those with a higher expression level in upper molar. We also split genes that were found to be differentially expressed at 1, 2, …, up to all (7) periods. **b** Distribution of temporal shifts of the upper/lower expression ratios, extracted by a method based on hidden Markov model. The size of the spots is proportional to the number of genes for which the upper/lower expression ratio follows a particular kind of profile. For instance, small–large at 14.5–15 indicates that the upper/lower expression ratio was small at ED 14.5 and larger at stage ED 15. **c** Classes and number of genes assigned to 8 temporal profiles for the genes that are differentially expressed at ED 15.5–16.0 but not earlier or later during the developmental series

We noted on the PCA plot (Fig. 2a) that the distance between lower and upper samples on axis 2 is maximal at ED15.0–16.0 (40–60% more than the average distance), which is also true when differentially expressed genes are removed. This suggests that some of the gene expression differences driving axis 2 are exacerbated during this period. A simple plot of the number of differentially expressed genes along development (Fig. 4a) also shows such transitory excess. “Upper genes” (genes with a significant positive upper > lower difference) are overall more numerous than “lower genes,” but the underlying distributions are different for genes differentially expressed at a single time point and for other genes. Finally, for genes differentially expressed at all time points, “lower genes” are very rare compared to “upper genes.”

We also designed a method, based on hidden Markov Models, to track differences in time profile, in a way which explicitly models the temporal series (see “Methods”). This models, for each gene, the variation of the magnitude of upper/lower ratios along time. More specifically, we considered two states, one with “small” and one with “large” upper/lower ratio. After modeling, each gene corresponds to a succession of eight states, corresponding to the variation of upper/lower ratios during the time course. This kind of modeling has a property of smoothing the time profiles (because the state probabilities in consecutive stages are not independent), rendering the results robust to artifactual changes of expression in a particular time point. In Fig. 4b, we counted the transitions for each pair of consecutive stages in our time series, that is, how many genes switch from one state of upper/lower ratio to another. Although the whole modeling is symmetrical along time, the resulting pattern is clearly asymmetrical (Chi-squared test, *P* value <10-16). Most transitions “small”–“large” were found between the first two time points, and on the contrary most transitions “large”–”small” were found between stages ED16 and ED16.5. This again emphasizes that upper/lower differences were time dependent, with a transient excess of upper/lower difference.

To understand what drove this transient excess, we retrieved 739 genes that were differentially expressed between lower and upper molars at ED15.5–16.0 but not at other time points (adjusted *P* value < 0.1 when both time points are considered replicates and significant in no more than one other time point). These genes were enriched for genes involved in mitotic cell cycle process (regulation but also accomplishment, e.g. formation of spindle…), but also in cell adhesion and cell migration (notably integrin binding), signaling (notably Wnt signaling pathway, with three ligands and two antagonists), ossification-related process, and programmed cell death. We classified these genes as lower or upper genes (Fig. 4c) and compared their relative enrichment for GO processes and functions. Lower genes (and most notably the 200 genes transiently downregulated in upper tooth, Fig. 4c) were strongly enriched in genes involved in cell cycle and mitosis (e.g. “cell cycle process” 5.10–57; spindle organization 1.10–18). This was reminiscent of the enrichment seen in upper cluster 1 (see above Fig. 2b) and again pointed an exaggerated decrease in proportion of mitotic cells in the upper molar germ at ED15.0–16.0. Conversely, upper genes were enriched in genes involved in cell migration/locomotion.

### Origins of the transcriptomic delay between the two molars

#### The transcriptomic delay of the upper molar is evidenced with several methods

**Fig. 5:**
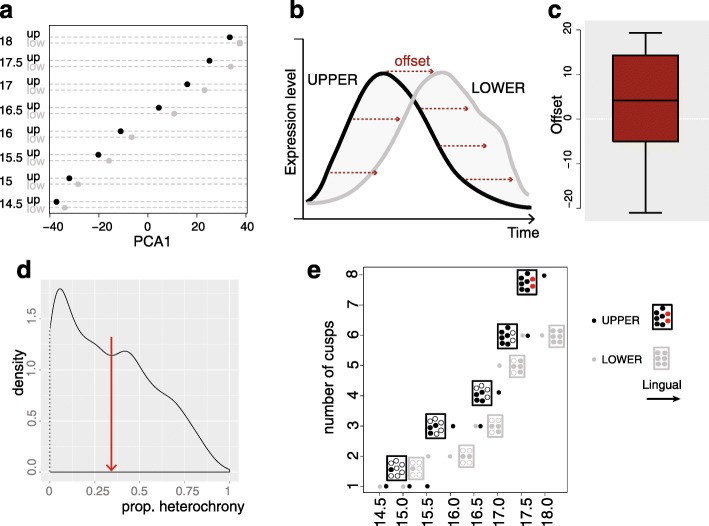
The heterochrony seen at the transcriptome level is not directly consistent with the number of patterned cusps. **a** Coordinates of the samples on PCA1 showing a systematic offset between upper (*black*) and lower (*gray*) samples, the latter being more “mature” than the former. **b** *Heterochrony measured on bell-shaped genes*: toy example showing how, as a proxy for heterochrony, we are using the simple time shift from upper to lower. **c** Distribution of this time shift, which is significantly positive, indicating that the upper molar germ is most often delayed in comparison with the lower germ. **d** *Proportion of the upper–lower distance, which can be explained by a simple time-translation*. The average is indicated by a *red arrow*. **e** Total number of patterned cusps at each developmental stage for upper (*black*) and lower (*gray*) molar of the RNA-seq time series. At each stage, embryos of the same litter and same weight as those chosen for the RNA-seq time series in panel (**a**) were used for in situ hybridizations with a Fgf4 probe marking the SEK

We noticed that the first axis of the PCA also tends to segregate lower and upper samples, although in a less intuitive manner than the second axis, since upper samples mapped systematically ahead from their lower counterpart (Fig. 5a). Because the upper and lower tooth at each developmental stage were taken from the same embryo, this cannot be due to sampling effect and rather suggests that upper tooth germs show a delay in shared developmental variation of gene expression. For instance, at ED 15 we estimated the offset between the two teeth worth 3.5–7.5 h of development (from simple ratio of the distances on the first axis, with consecutive development stages in upper or lower tooth being estimated at about 12 h). We later refer to this delay as an “heterochrony” between upper and lower tooth development. We then wished to estimate this heterochrony using a completely independent method, based on expression profiles. It is not possible for genes with a linear profile of expression to disentangle shifts in expression levels (heterometry) and shifts in expression timing (heterochrony) on an individual basis. Nevertheless, on a statistical basis, if the upper germ is delayed, then linear genes increasing with time should tend to be more strongly expressed in lower germ whereas those decreasing should tend to be more strongly expressed in upper germ. This was indeed the case when taking genes that vary significantly during development, in a linear manner (comparison of 3281 genes whose expression level is increasing linearly, with 3181 genes which are decreasing linearly: Wilcoxon test, *P* value < 10-16; DE genes with a significant time-related bias, adjusted *P* value < 0.05, DESeq2).

To go further, we decided to focus on genes for which the expression profile is a bell curve, with a single extremum of the same kind (either a maximum or a minimum) (Fig. 5b). We found 1029 such genes. We then time-shifted the expression profile for the upper tooth, so that to match the lower profile as well as possible (using a simple translation, see “Methods”). The distribution of the translation factors was significantly skewed towards positive values, in accordance with the hypothesis that the upper tooth is overall delayed by comparison to lower tooth (Fig. 5c). We then sought to quantify the relative importance of heterochronies in the upper–lower difference. For that purpose, we quantified the upper–lower distance before and after time-translation. The ratio indicates that, on average, 32% of the difference between upper and lower (bell-type) profiles is attributable to simple heterochronic shift (median = 29%, Fig. 5d). We concluded that a substantial part of gene expression differences (one-fourth to one-third) between lower and upper tooth germ can be interpreted as delayed changes in gene expression in the upper molar.

**Fig. 6:**
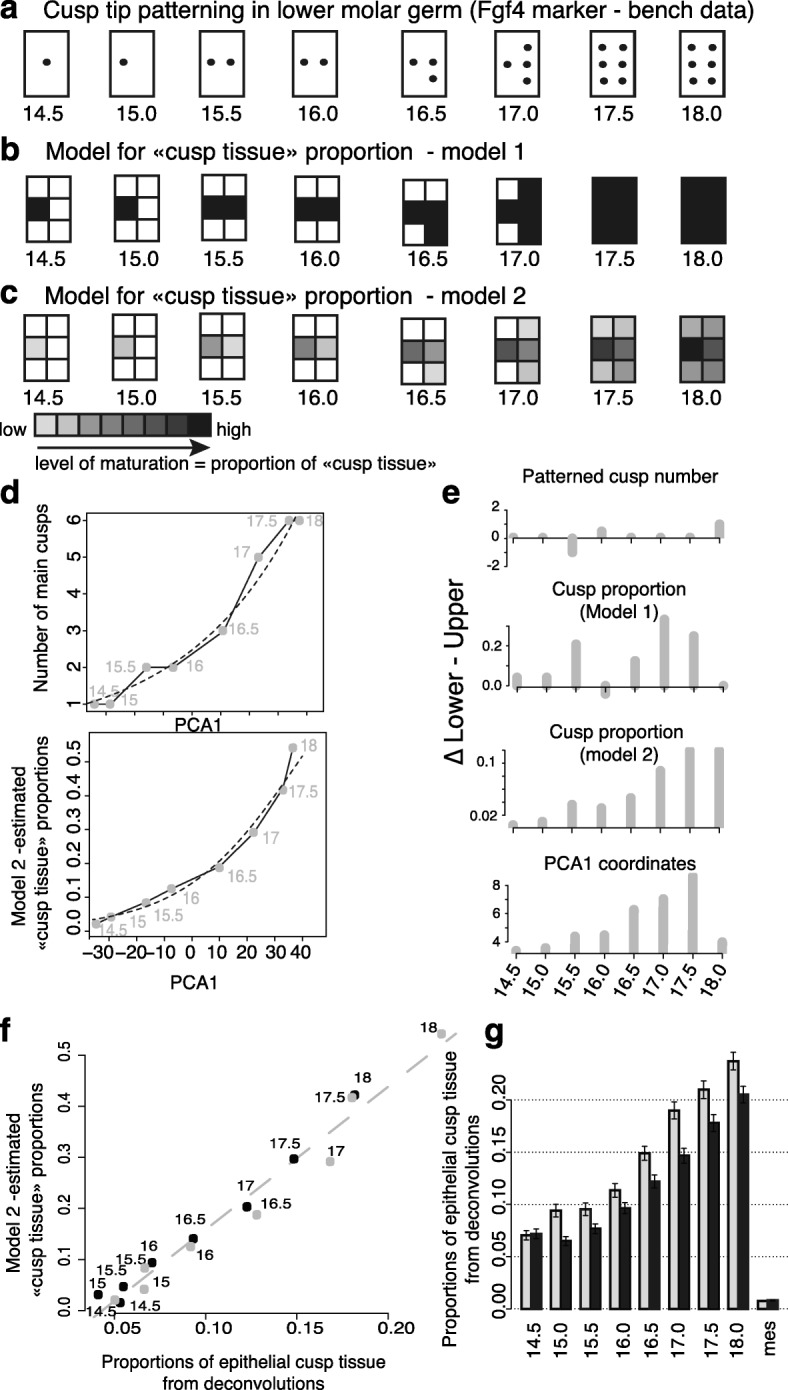
Cusp proportions estimated with a simple model recapitulate time ordering on first PCA axis and upper–lower heterochrony. **a** Cusp patterning in the lower molar, from one to six main cusps, which corresponds to its adult morphology. The timing and sequence of cusp addition reflects observations based on the analysis of in situ hybridization data using the cusp tip marker Fgf4 (see “Methods” and Fig. 5e). **b** Model of cusp proportion for the lower molar without maturation. The contribution of each cusp to a final proportion of “cusp tissue” (represented by the last stage) is modeled with a simple grid. In this model, the total contribution is achieved as soon as the cusp has been patterned. Time for cusp patterning is derived from bench data in (**a**). **c** Model of cusp proportion for the lower molar with maturation. In this model, the contribution of each cusp to a final proportion of “cusp tissue” increases following patterning. “Cusp tissue” expands at the same speed in all cusps. The average shade of gray of grid unit, at each developmental stage, gives a visual impression on the average degree of the expansion of the cusp tissue territory, from unpatterned cusp (*white*) to just-patterned cusp (*light gray*) and fully expanded cusp (*black*). We computed cusp expansion in each tooth based on this model, 0 corresponding to no cusp patterned (*all white*), and 1 to a complete expansion (*all black*). **d** *Top*: Relationship between the number of cusps, as shown in (**a**) and the coordinate on the first PCA axis (developmental stages are indicated). *Dashed lines*: glm model in which PCA1 explains cusp number (corresponding *P* value = 0.003). *Bottom*: Relationship between cusp proportions, estimated using model 2 (shown in (**c**)), and the coordinate on the first PCA axis (developmental stages are indicated). *Dashed lines*: glm model in which PCA1 explains cusp proportion (corresponding *P* value = 1e-12). **e** Lower/upper difference as estimated in terms of cusp number (as in (**a**)), cusp proportion estimated with model 1 in (**b**), cusp proportion estimated with model 2 in (**c**), and coordinates on PCA axis 1. Model (**c**) recapitulates best the heterochrony measured on PCA1. **f** Relationship between the proportion of “cusp tissue,” estimated using the maturation model in (**c**), and the proportion of “epithelial cusp tissue” estimated by deconvolutions. Six markers from bite-it (*Bmp7*, *Edar*, *Fgf9*, *Cdkn1a*, *Wnt10b*, *Shh*) were chosen, because their expression territory is large and therefore may well reflect the advancement of cusp expansion. **g** Proportions of “epithelial cusp tissue” estimated by deconvolution as in (**f**) for upper (*black*) and lower (*gray*) samples, at each developmental stage. RNA-seq samples of mesenchyme isolated from lower and upper molar germs [45] serve as negative controls (“mes”)

The period of interest is marked by crown morphogenesis, including cusp sequential addition and the initiation of their differentiation. Heterochrony in this process could thus logically result in transcriptome heterochrony, and especially along PCA1, since, as seen earlier, EK marker genes are among strong contributors to this axis. To try to understand this heterochrony in expression, we first examined the link between incremental cusp patterning and PCA1. We documented the temporal dynamics of cusp addition in the two molars, using the cusp tip marker Fgf4 (in situ hybridization data; see Fig. 6a for lower and Additional file 1: Figure S5 for lower/upper data). The dynamics is clearly different between upper and lower molars (Fig. 5e). However, from these data, one does not intuitively expect a systematic delay of the upper molar, like the one seen in expression data, which starts from early stages and continues throughout the development.

#### The transcriptomic delay between the two molars relates to the proportion of “cusp tissue”

To better understand this pattern of upper/lower heterochrony, we needed first to better understand the link between PCA1 and the number of cusps for one tooth germ, for example the lower one. The number of cusps that are patterned at each developmental stage was correlated with the coordinate on PCA1 (Fig. 6d top: *P* value associated to a Poisson model in which PCA1 explains cusp number = 0.003, McFadden’s pseudo R-squared = 0.29). This is a good correlation, of course, but the number of cusps does not increment at each developmental stage (e.g. between 14.5 and 15.0 or between 15.5 and 16.0) and yet the transcriptome captures time differences between those stages. This is not surprising as correlating with absolute cusp number is over simplistic for two reasons. First, a transcriptome made from a whole tooth germ can only record a proportion of tissue engaged in cusp formation relative to the rest of the tooth germ and not absolute number of cusps. Second, cusps and in particular cusp epithelium, are known to mature progressively from tip (the so-called EK, see Fig. 1) into the valleys (starting with cell cycle arrest and much later with ameloblast differentiation) and this process starts soon after the EK are patterned [54]. We thus anticipate that the patterning of EK should be very rapidly followed by conversion of naïve tissue to “cusp tissue” from the tip into the valleys and this would also change cusp proportions.

Based on these assumptions, we built a simple model for quantifying “cusp tissue” patterning and expansion in time, fueled with our *Fgf4* in situ hybridization results on time of cusp tip (SEK) patterning. For simplicity, we chose to model cusp proportions with a simple grid model. This is still very simplified as there is no pre-pattern in tooth and cusp patterning occurs concomitantly with some tooth growth. For simplicity, also, once patterned, the territory of each cusp expands at the same speed (Fig. 6c). Despite this simplicity of the model, its application to lower tooth fits better the advancement of development monitored by PCA1 than the simple number of cusps, albeit in a non-linear manner, which was expected given that we estimate proportions (Fig. 6d bottom; *P* value associated to a binomial regression in which PCA1 explains cusp proportion = 10-12, McFadden’s pseudo R-squared = 0.42). This shows that the overall proportion of the territory occupied by cusp tissue, very simply explains the major component of the transcriptomes.

We then sought to understand whether this main model could explain the offset observed on the PCA1 between upper and lower tooth. We applied a similar modeling to the upper tooth, following the sequence of cusp addition that ranges from one to eight cusps, because the upper tooth adds two more cusps at the end of the developmental sequence (ED17.5–ED18.0, Fig. 6e and Additional file 1: Figure S5a; *P* value associated to a binomial regression = 10-24, McFadden’s pseudo R-squared = 0.71). We fueled it with *Fgf4* in situ hybridization data obtained for upper molar (see “Methods”), since the dynamics of cusp patterning is specific to each molar (see above Fig. 5e). As such, the model intrinsically implies that in the early steps, the cusp tissue will occupy a smaller proportion of the total tooth germ in the upper tooth as compared to the lower tooth and also that cusp expansion rate is similar between the lower and upper molars. This may not be realistic and therefore we examined three alternative models (Additional file 1: Figure S5b–d): a first model, in which the two supplementary cusps are omitted (implying that the formation of extra cusps does not translate in a change in cusp proportions); a second model, in which the upper tooth is progressively acquiring the extra space corresponding to the extra cusps; and a third model, in which the rate of cusp expansion is different in upper as compared to the lower tooth. With respect to the overall fit to PCA, none of these alternative models performs better than the main model and they are all worse considering specifically upper/lower heterochrony (see details in the legend of the Additional file 1: Figure S5).

The offset between upper and lower tooth at the same stage on the PCA (which we called earlier heterochrony) is well correlated with the offset of cusp proportions computed from the model (R2 = 41%, Fig. 6e, model 2). This correlation becomes nearly perfect (R2 = 95%, *P* value = 10-4) when the last stage (ED 18) is omitted. This suggests that our model, in which cusp expansion is infinite, is not adequate at this late stage, possibly because cusp expansion is in fact finite. We modified our model in accordance with this hypothesis, by reducing the time for complete expansion attributed to each cusp. We reduced this time, from eight steps (corresponding to the entire time series, therefore to the non-finite expansion model mentioned above) down to two steps. The pattern of heterochrony observed with the PCA is best recapitulated by a finite model in five steps, i.e. which allows a duration of 2.5 days for each cusp to expand to its final territory (Additional file 1: Figure S5e).

A simple model of “cusp tissue” proportions in upper and lower molar germs was thus sufficient to recapitulate the main axis of variation observed at the transcriptome level and the heterochrony between the two molars. However, at this stage we did not make explicit what could be this “cusp tissue” whose expansion impacts the transcriptomes. In theory, both the epithelial and mesenchymal component may be concerned but at least this “cusp tissue” should include the SEK and the neighboring epithelial tissue, which gets patterned soon. This process is exemplified by *Shh* expression, which is stronger in the SEK but spread in a much larger territory of dental epithelium around ([55] and http://bite-it.helsinki.fi/ [52]). In the bite-it database, we screened for other genes which expression territory is first confined to the PEK (cap stage) and later (bell stage) expanded to a larger epithelial territory around the SEK. We thus defined six markers of what we refer below as “epithelial cusp tissue” (*Shh*, *Bmp7*, *Edar*, *Fgf9*, *Cdkn1a*, *Wnt10b*).

With this set, we performed deconvolutions to monitor the progression of epithelial cusp tissue proportion in lower and upper molar germs. As expected, the proportion is increasing with developmental stage in upper and lower tooth and epithelial cusp tissue proportions themselves are also very well correlated with PCA1 (*P* value = 10-8; R2 = 0.91; Fig. 6f). Epithelial cusp tissue proportions are comparatively smaller in upper than in lower molar germs (Fig. 6g). The upper/lower offset in the advancement of the maturation estimated by our model was nearly perfectly correlated with epithelial cusp tissue proportions estimated by deconvolutions (*P* value = 10-12; R2 = 0.97). The offset was small at the beginning of the developmental series, then tends to increase with developmental time, and is reduced again at the end. This further suggested that the upper germ transcriptome may look delayed because less “cusp tissue” has formed and therefore genes expressed in this “cusp tissue” were less represented in the whole tooth germ.

#### Mosaic heterochrony in different tissue compartments

A previous section of the paper suggested that most of the information concerning development time in the transcriptome was carried by “epithelial cusp tissue” proportion. Upper and lower heterochrony seemed also easily explained by differences in the advancement of “cusp tissue” proportion between the upper and the lower tooth. However, bell-shaped genes also show heterochronies and also order samples on PCA, albeit their bell profile is not directly explainable by a steady progress in cusp formation. As a result, we can imagine that time information may also be carried by other tissues, e.g. mesenchyme and non-cusp epithelium. To get hints into this question, we selected roughly the same number of marker genes for three tissues: EK (421 specific genes), epithelium (336 specific genes), and mesenchyme (566 specific genes). We then first asked whether each set of markers is carrying time-related information, that is, whether the three separate PCAs were made using each tissue-specific set of markers order samples on their first axis (PCA1). The answer is “yes” for each “tissue-specific PCA", although the percentage of variation associated to each of these first axes is varying. As expected, PCA1 is strongest for EK-specific PCA (made with EK markers, 68.4% of variation), but mesenchyme-specific PCA (57% of variation) and epithelium-specific PCA (50.6% of variation) also carry a strong time-related signal (Additional file 1: Figure S6). The three tissue-specific PCAs also carry a signal of heterochrony. EK-specific PCA carries a heterochrony (up to 15% of the total distance ED14.5–18) which is best correlated with the heterochrony measured on total PCA (63.0%, *P* value = 0.02) and, reassuringly, with differences in EK proportions measured with deconvolutions (63.0%, *P* value = 0.02). Epithelium-specific PCA carries very little heterochrony, but very interestingly, mesenchyme-specific PCA carries a very strong heterochrony (up to 40–50% of the temporal signal of mesenchyme). Contrarily to the pattern of heterochrony carried by EK, the pattern of heterochrony carried by mesenchyme is quite stable throughout the time period and starts in early development.

Using a trick of normalization (see “Methods”), we sought to disentangle the influences of tissue proportions and changes in cell types in time-related effects. As expected, time-related effects in EK seem to be carried both by tissue proportions and by cell-specific effects (PCA1: diminishes from 68.4% in non-normalized PCA to 59.5% in normalized PCA). In mesenchyme, the magnitude of time-related effects is a bit less impacted by normalization (PCA1: decreases from 62% to 57% for mesenchyme) and in epithelium there is hardly an effect (from 50.6% to 49.9%). This meant that temporal effects for in mesenchyme are not much carried by tissue proportion but rather mainly by tissue maturation (see also deconvolutions of mesenchyme proportion in Fig. 3b which are quite stable with time). From the same tissue-specific PCAs, we observed that the patterns of upper/lower heterochrony in EK and mesenchyme stand against normalization, which means that heterochrony is also carried by tissue maturation (Additional file 1: Figure S6).

We conclude that the transcriptomic delay of the upper molar seen from the earliest stage on is the result of at least two phenomena: (1) a smaller proportion of “epithelial cusp tissue",; and (2) a slower maturation of EK and mesenchyme tissues. An interesting possibility would be that this delay in mesenchyme maturation is linked with the patterning of two extra cusps in the upper molar at ED17.5–18.0 (Fig. 5e). This apparent delayed maturation might reflect a developmental mechanism whereby progression of differentiation is delayed, thus lengthening the phase when the germ grows and new cusps are patterned.

## Discussion

This study asks the question which transcriptomic differences distinguish the development of two serial organs with different morphologies: the lower and upper first molar of mouse. We identified a shared developmental program despite heterochronies (temporal shifts) in this program. These are common findings in comparative developmental biology, but usually made from a limited number of genes. Here we show this is mirrored in the whole transcriptome dynamics. Furthermore, we revealed several collective gene expression differences, which we call transcriptomic signatures. First, we evidenced a transcriptomic signature for lower–upper molar heterochrony that we could relate to differences in proportions of the tissue later forming the crown cusps. Second, we found a transient signature due to exaggerated expression profiles in the upper molar, early during crown molar morphogenesis. Related to this, transcriptomic differences did not accumulate as in a funnel model, but peaked early on during molar crown morphogenesis. Third, we evidenced a clear lower/upper transcriptomic identity carried by a very large number of genes, for which individual variation was generally small but consistent over developmental stages. This signature stemmed from upper/lower biases in tissue composition of the tooth germs, although expression within individual tissues (at least mesenchyme) also participated.

Below we discuss how these transcriptomic signatures highlight variations in a core developmental program that are possibly relevant for final phenotypic differences and evidence properties of whole organ transcriptomes applicable beyond our specific biological context.

### Transcriptomes reveal a core developmental program modified by cell proportions, heterochronies, and inverted hourglass variation

It is commonly thought that serial organs develop using similar gene networks (e.g. limbs [18, 20]). We already knew that the two molars express more or less the same genes [28]. By showing that more than half of those genes have very similar temporal dynamics, we demonstrate that the two molars use similar gene networks, with similar temporal deployment. This identifies a molar core developmental program. Of note, some authors propose this is a criterion to establish serial homology [56]. This would replace the strict criterion of common ancestry, which implies that in a distant ancestor, serial organs were identical and sharing identical gene networks. This is generally difficult to establish with certainty [57], exactly the case for molars [21]. According to the extended definition of serial homology, our finding of a core molar developmental program establishes that lower and upper molars are serial homologs.

**Fig. 7:**
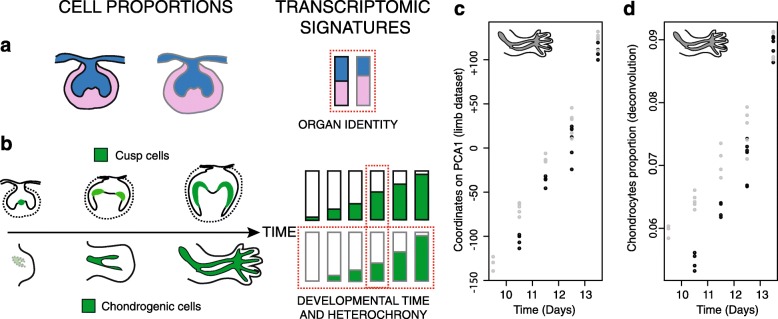
Transcriptomic signatures shaped by cell proportion reveal differences in organ development. **a** A difference in mesenchyme (*pink*) versus epithelium (*blue*) proportion in the tooth germ takes part in a transcriptomic signature of organ identity. **b** Increasing proportion of differentiating cells over time takes part in a transcriptomic signature of developmental time and heterochrony. We evidenced this both in molars (cusp cell differentiation) and limb (chondrogenic cells, see (**c**) and (**d**)). **c**, **d** We reanalyzed a published transcriptome dataset on forelimb/hindlimb development obtained with microarrays by [27]. On the first axis of PCA, we recovered a strong time-related signal (**c**) and the expected heterochrony between forelimb and hindlimb (forelimb samples are systematically ahead on PCA1 (**c**)). Both the time signal and the heterochrony correlated well with the proportion of chondrogenic cell types estimated by deconvolution with a set of marker genes extracted from [102] ((**d**), compare (**c**) and (**d**))

In contrast to these shared aspects, the final differences in upper/lower morphology imply that the two molar germs exhibit extensive differences during morphogenesis. We evidence a clear genomic signature of lower/upper identity, consistent throughout very different periods of molar development (bud to end bell stage). We dissected this genomic signature and showed that this identity is influenced both by the proportion of the different tissues composing the tooth germ (epithelium–mesenchyme) and the tooth-specific nature of tissues (at least for mesenchyme, since this was not specifically examined for epithelium). A recent study evidenced that organ-specific transcriptomic signatures in recently specified organs can be accounted for by a limited number of master transcriptional regulators, specifically expressed in the organ and activating a vast amount of target genes [7]. A similar effect may be at work here and could rely on the few specific or markedly biased transcription factors already known or identified here (e.g. Nkx2.3, reported previously in molars [42], Pou3f3 and two genetically linked ncRNA (2610017I09Rik and 2900092D14Rik, reported previously only in early jaws [50], or the identity genes Dlx5 and Dlx6 that are no longer specific for lower molar but expressed two to three times less in upper molar). This could be further investigated in the future. It remains that most of the transcriptomic identity is made by differences in mesenchyme proportion within the germ (Fig. 7a). We revealed this by transcriptomes analyses and then confirmed it at the bench: we quantified the respective volumes of the mesenchyme/epithelial compartments in 3D reconstructed tooth germs (dissected with the same procedure as the RNA-seq samples, Additional file 1: Figure S4) and the respective areas of the two compartments delineated on histological sections (which rules out the possibility that it could be a dissection artifact). The upper molar showed a larger proportion of mesenchyme from ED14.5, a very early step for crown morphogenesis. This finding is interesting in light of a tooth engineering study [58]. When an isolated ED14.5 epithelium was allowed to develop on a pellet of dissociated mesenchymal cells, tooth and cusp formation were obtained and the number of cusps formed increased with the number of mesenchymal cells. A provocative scenario could be that the excess of mesenchymal cells present since ED14.5 in the upper molar is sufficient to raise the levels of cusp-inducing molecules secreted by the mesenchyme (such as Activin [55]) and favors the formation of additional cusps. In this case, tissue architecture, without any need for differential gene regulation within cells during this period, would be one determinant of upper–lower differences. It is ironic that cell proportions play a major role in shaping the genomic signature of lowerness/upperness, such that our study initially focused on genes reminds us not to set cells aside: a view centered on cascades of gene expression is insufficient, because both molecular and cellular differences feed off one another over the course of development.

Another obvious factor well known in comparative developmental biology is the difference in the relative timing of developmental processes (heterochronies). Heterochronies have been repeatedly pointed out in studies comparing serial organ development (and more generally in comparative developmental biology) and to date have somehow impeded the comparison of transcriptomes between serial organs [59]. Our data are consistent with this as heterochronies explain a substantial part of expression profile differences (25%). We moreover evidenced a transcriptomic signature of heterochrony: the upper molar is systematically “younger". To our knowledge, no study has ever reported a delay in the upper molar development during the period we examined here. Nevertheless, it is consistent with a delay reported for stages older than our oldest timepoint, in the doctoral work of Moullec [60]. He showed that odontoblast differentiation and enamel secretion, two processes that start at end of the fetal period, become visible one day later in upper molar compared to lower molar. Moreover, it would not have been expected from standard comparative developmental biology data (see our *Fgf4* data, Fig. 5). The transcriptomic delay is seen from ED14.5, before cusp addition starts. Importantly, it is not due to a constant offset in the timing of development of the two molars, since cap transition, which is seen at late ED14.0, is synchronous in both molars (data not shown). It is thus far from trivial. Nevertheless, the heterochronic shift measured at each timepoint was recapitulated in a simple model predicting proportions of cells engaged in cusp formation from timing of cusp formation estimated at the bench with the *Fgf4* marker. According to this model, from ED14.5, the proportion of cells engaged in cusp formation is smaller in the upper molar than it is in the lower molar. This finding suggests a mechanistic difference in cusp formation between the two molars, possibly in relation with the formation of two additional cusps in the upper molar. Cusps are known to inhibit the formation of new cusps in their vicinity [34]. From early on in crown morphogenesis, a decreased cusp proportion will reduce the inhibition on the unpatterned part of the tooth germ. This mechanism may promote the upper molar making ultimately more cusps. This could be achieved by change(s) in the growth rate and/or activation–inhibition mechanisms that together rule cusp addition [34, 61]. Heterochronies are further discussed in the last section.

We expected the temporal structure of expression differences to culminate in the latest stage. This intuitive idea is integrated in two models of interspecific variation in embryogenesis: the funnel-like model where differences in gene expression increase linearly; and the hourglass model where differences are minimal at mid-embryogenesis but increase afterwards (e.g. [11, 12, 62–65]). In our case, we had additional reasons to expect such a pattern. The supplementary cusp row of the upper molar starts forming at a time when all main lower cusps are patterned (ED17.5, Fig. 5d). It follows lingual extension of the upper germ beyond ED16.5, at a time when this process has stopped in the lower germ [41]. This prolonged duration in the upper molar should logically emphasize differences with the lower molar late in development. However, all transcriptomic data analysis (PCA, DE genes, Markov modeling) pointed to an early peak of differences (ED15.0–16.0), in conflict with the funnel-like model. The transcriptomic variation follows what we call an inverted hourglass model. This observation suggests that differences in developmental processes can be much sharper in early stages than commonly thought and can peak before partially resuming.

Why then such an early peak of differences? This ED15.0–16.0 period may be critical for the phenotypic differences between the two molars. The sharper gene expression profiles seen for upper molar at ED15.0–16.0 indicated that it experiences exaggerated processes (see Fig. 2). Interestingly, genes differentially expressed at this period included genes involved in cell adhesion and cell migration, suggesting that in the upper molar, a higher proportion of cells are engaged in these processes. This is consistent with intense morphogenetic changes in the epithelium, but also in mesenchymal cells that are proliferating, migrating, and segregating to form the dental papilla and the dental follicle [66, 67]. Moreover, transcriptomes also showed that molar germs in this period are slightly depleted in mitotic cells and this effect is marked in the upper molar. This depletion may also reflect the intense morphogenetic changes at this stage, as a dichotomy between cell division and cell shape change has been observed (in cancer cells [68], during embryogenesis in various species [69]).

Independent of the transcriptomic analysis, our bench data are consistent with differences from this early period, as we see delayed patterning of the second cusp in the upper molar (Fig. 5d). As the two molar germs enter morphogenesis (“cap stage”) synchronously, just before ED14.5 (Fig. 1), we have to assume that the upper molar takes more time before the second cusp is patterned. Interestingly, this cusp is patterned following the rapid lingual extension of the molar germ and the persistence of this process in the upper molar enables the formation of a third cusp row [41]. It is possible that morphogenetic processes linked with the lingual development of the molar germ are exaggerated in this ED15.0–16.0 period and prepare for the later formation of the supplementary cusp row. Such a mechanism for supplementary cusp formation is not mutually exclusive with the difference in activation–inhibition mechanisms proposed above. Both types of evidence concern an early period of crown morphogenesis, while the formation of the third cusp row occurs at the very end of morphogenesis: we thus suggest a scenario where early variation in a core molar program is responsible for ultimate phenotypic differences.

### A general approach of transcriptomes for evolutionary developmental biology and developmental biology

Beside our biological question, our results evidence properties of whole organ transcriptomes with strong implications for developmental and evolutionary developmental biology. Transcriptome comparisons are commonly used in both fields, whether it be between mutant and wild type, between species at specific timepoints (including adulthood), or between stages in a specific time course.

First, we show that transcriptomes allow determining the stage of an organ, even at a fine scale. This self-ordering of developmental time series in a multivariate analysis and its convenience for blindly staging samples has been demonstrated in other datasets (e.g. [6, 70]). Our dataset represents a much more restricted portion of the development, because it is sampled at the level of the organ and not at the level of the whole organism, during a tight window of development. The self-ordering of developmental samples appears because a sufficient number of genes show variations that are subtle, yet consistent between consecutive stages.

Second, we show that cell proportions shape clear signatures in the transcriptome of a whole organ (Fig. 7a, b). This is true comparing different organs (mesenchymal versus epithelial cell proportions) or different developmental stages of the same organ (proportions of mitotic cells or cusp forming tissue). Importantly, this finding was not limited to molar germ development: by analyzing the most comprehensive dataset of forelimb and hindlimb development [27], we recovered a strong time signal on PCA1 that correlated well with proportions of chondrocytes estimated by deconvoluting 50 key marker genes (Fig. 7c, d and Additional file 1: Figure S7 and supplementary text).

The signature can be a matter of a proportion of cell types per se (like we see in the case of epithelial versus mesenchyme, cusp tissue, or chondrocyte proportion), or a matter of a proportion of cells, engaged in different parts of the cell cycle, regardless of cell type. The effect of cell cycle activity on the heterogeneity of gene expression is reminiscent of the major impact seen in single cell RNA-seq data, where it is often treated as a confounding factor [71–73]. In batches of yeast cells, the proportion of cells that stand at different time of the cell cycle is also largely impacting the transcriptomes [74]. To our knowledge, this is the first time such an effect was interpreted in a comparison of complex organs, even if it is easily conceivable that gene expression of a whole organ reflects its cellular composition.

In summary, changes in gene expression taken at the transcriptome level reflect: (1) the restriction and expansion of cells populations, each expressing a given repertoire of genes; (2) the appearance of new “cell types” expressing radically different repertoires of genes; and (3) above that, the variations in proportions of cells found in different phases of the cell cycle. This view should be applied to many other types of transcriptome comparisons common in developmental biology and evo-devo. In developmental biology, it is common to compare, at a given timepoint, the same organ in a wild-type situation versus a mutant situation. Our findings suggest that the transcriptomic changes provide a monitoring tool of the expansion of some cellular populations to the expense of others (e.g. in mutants with a patterning defect). It is now common to compare the transcriptomes of different species (developing or adult organs) [12, 75–77]. Here, transcriptomic differences may reveal the physiological adaptations of an organ resulting from the relative expansion of a given tissue or cell type within the organ. Combining whole organ transcriptomes with tissue or cell-specific transcriptomes is a powerful approach to get an integrated description of developmental gene expression.

Finally, transcriptomes reveal heterochronies, a key source of differences between species [78]. A few studies have reported transcriptomic heterochronies [79–83]. On the other hand, many “classical” heterochronies have been reported in serial organs, most notably between mammalian limbs, based on morphological criteria or marker genes [25, 26].

To our knowledge, our study is the first report of a transcriptome heterochrony at such a fine scale (a few hours for molars) and between developing serial organs. Importantly, our approach applied to another dataset also recovered the expected heterochronic signal: the forelimb was slightly advanced compared to the hindlimb, as expected (Fig. 7c, d and Additional file 1: Figure S7 and supplementary text). We showed that the proportion of differentiating “cusp-tissue” for molars or differentiating chondrocytes for limbs (Fig. 7d) increases with time. The relatively smaller proportion of cusp tissue in the upper molar or chondrocytes in hindlimbs makes them look younger.

In molars, transcriptomic heterochrony associated with cusp epithelium is a matter of cell proportion (Fig. 7b). In addition, we also see that mesenchyme displays its own pattern of heterochrony: whole tooth heterochrony is most likely the result of different patterns. As pointed out by Gould [78] and others [84], heterochronies are most often patterns and only rarely a direct mechanism for phenotypic difference. Yet identifying heterochronies may help to formulate hypotheses, as seen above.

The detection and elucidation of transcriptomic heterochronies will be a very helpful tool to gain insights into the developmental basis of species differences. We anticipate that in any system, where the proportion of a given cell type changes over time, a difference in that proportion between organs will also be perceptible as a transcriptomic heterochrony (independently of technology, as we employed here both RNA-seq (molars) and microarrays (limb) technologies).

## Conclusions

Our study extends the use of transcriptomes as molecular phenotypes by enhancing our comprehension of the transcriptomic patterns that are reported. We evidenced three transcriptomic signatures, all stemmed from differences in cell proportions between organs and stages. From these, we could formulate specific hypotheses how two serial organs develop in two different morphologies. We predict that transcriptomes taken as “molecular microscopes” will reinvigorate comparative embryology [2]. More generally, we believe transcriptomic signatures in complex organs will help deciphering biological variation in a variety of contexts, from developmental biology to evolutionary biology or medical science (see also [2, 70]).

## Methods

### Mice breeding and embryo sampling

CD1 (ICR) adult mice were purchased from Charles River (France). Females were mated overnight (at 20:00) and the noon after morning detection of a vaginal plug was indicated as ED0.5. Other breeding pairs were kept in a light-dark reversed cycle (12:00 midnight), so that the next day at 16:00 was considered as ED1.0. Pregnant females were killed by cervical dislocation and embryos were harvested on cooled Hank’s or DMEM advanced medium, weighted as described in [85] and immediately decapitated. This study was performed in strict accordance with the European guidelines 2010/63/UE and was approved by the Animal Experimentation Ethics Committee CECCAPP (Lyon, France; reference ENS_2012_045).

### Epithelium dissociations and in situ hybridizations

Complete or hemi mandibles and maxillae were dissected in Hank’s medium and treated with Dispase (Roche) 100% at 37 °C for 1 h 30 min to 2 h 20 min depending on embryonic stage. Epithelium was carefully removed and fixed overnight in PFA 4% at 4 °C. DIG RNA antisense *Fgf4* probe were prepared from plasmids described elsewhere [86]. In situ hybridization was done according to a standard protocol. Photographs were taken on a Leica M205FA stereomicroscope with a Leica DFC450 digital camera (Wetzlar, Germany) or on a Zeiss LUMAR stereomicroscope with a CCD CoolSNAP camera (PLATIM, Lyon, France).

### RNA-seq sample preparation

A total of 16 samples were prepared for the RNA-seq analysis, representing eight stages (ED14.5, 15.0, 15.5, 16.0, 16.5, 17.0, 17.5, 18.0), coming from eight individuals. Each sample contained two tooth germs, the left and right first molars (M1) of the same male individual, and for a given stage, the upper and lower samples came from the same individual. The heads of harvested embryo were kept for a minimal amount of time in cooled PBS (small scale) or advanced DMEM medium (large scale). The M1 lower and upper germs were dissected under a stereomicroscope and stored in 200 uL of RNA later (SIGMA). Similarly dissected tooth germs from the same litter and same weight were fixed overnight in PFA 4% for immunolocalization and 3D reconstruction (see later). Another embryo of the same litter and same weight was processed as indicated above for *Fgf4* in situ hybridization. Total RNA was prepared using the RNeasy micro kit from QIAGEN following lysis with a Precellys homogenizer. RNA integrity was controlled on a Bioanalyzer (Agilent Technologies, a RIN of 10 was reached for all samples used in this study). PolyA+ libraries of the large-scale dataset were prepared with the Truseq V2 kit (Illumina), starting with 150 ng total messenger RNA and reducing the amplification step to only 12 cycles and sequenced on an Illumina Hi-seq2000 sequencer at the GENOSCOPE (Evry, France).

### Data analysis

R scripts corresponding to the main methods and processed data are available on GitHub (https://github.com/msemon/ToothTranscriptomeAnalyses) and in zenodo (doi:10.5281/zenodo.197077). Source code is free software governed byCeCILL License.

### Expression levels estimation using RNA-seq and differential expression analysis

Sequences were obtained for each sample (Illumina sequencing, 100 bp paired-end sequences). These reads were mapped to the mouse genome (mm10, October 2012), using Bowtie2 (version 2.0.2; [87]) and Tophat2 (version 2.0.6; [88]) with standard settings. We obtained a median of 37.8 million reads that mapped uniquely to the genome (minimum = 32.9 to maximum = 44.3 M reads). The number of reads per gene was then counted using HTSeq (version 0.5.3p9; option: “intersection-nonempty;” 21,938 genes with a UCSC annotation; [89]). Raw data are available in the gene expression omnibus repository. (GSE76316). Published RNA-seq data were uploaded (accession number GSE39918 and GSE36863) and treated as our own data for read mapping, counting, and normalization.

Statistical analysis was performed using R (version 3.1.2, 2014-10-31; [90]), with ggplot2 for graphics (ggplot2_1.0.0; [91]). Two main analysis used in the text include differential gene expression analysis and multivariate analysis. DE analysis was performed using DEseq package (DESeq2, version 1.6.3 [92]), with settings indicated in the main text. Multivariate analyses (mainly PCAs) were performed using ade4 package (ade4_1.6-2; [93]).

### Gene Ontology analysis

Gene ontology (GO) analysis was performed and visualized with GORILLA [94] and GOStats (version 2.34.0, with Mmusculus.UCSC.mm10.ensGene_3.1.2 and GO.db_3.1.2; [95], using the full list of genes expressed in the corresponding dataset as a background. R custom scripts were used for graphical representation of the GO-terms enrichments.

### Clustering of upper and lower time series

For each gene and at each stage of development, we took the level of expression divided by the initial level of expression (i.e. level at stage ED14.5). Following [96], we then ranked the genes according to the maximum obtained value and removed the first quartile (relatively flat genes), obtaining a set of 17,506 remaining genes. To classify the genes, we first generated theoretical profiles representing all possible combinations of transitions between stages (expression level being either increasing, decreasing, or flat between the eight consecutive stages), making up a total of 2187 profiles. We then correlated each real expression profile to each theoretical profile using Spearman correlations. We clustered the obtained correlation matrix using K-means (choosing ten clusters and checking that the obtained profiles are repeatable over several iterations of the method). The median of the expression profiles of these ten clusters are drawn on Fig. 2b.

### Simulations of realistic tooth transcriptomes with different tissue proportions

To simulate realistic tooth transcriptomes while controlling the relative proportions of mesenchyme (M), enamel knot (K) and non-enamel knot (E) epithelium, we used expression levels in isolated tissue obtained from microarray data, taking the median of ED12.5, ED13.0, ED13.5 ([39], http://compbio.med.harvard.edu/ToothCODE/). We consider that for each gene G, expression level is proportional to a mixture of its microarray levels (denoted respectively mMG, mKG, and mEG, respectively) in the three tissues. If we set the hypothesis that lower tooth is a mixture of pE epithelium tissue and (1-pE) mesenchyme, and that epithelium tissue is a mixture of pP non-enamel knot epithelium and (1-pP) enamel knot, then gene G specific expression proportion factor equals:

$$ k G= L G/\left(\left(1- pE\right)* mMG+ pE*\left( pP* mEG+\left(1- pP\right)* mKG\right)\right) $$

where LG is this gene expression level is the RNA-seq library of the lower tooth.

From this, we can simulate artificial RNA libraries with several tissue proportions, such as pE' epithelium and pP' non-enamel knot in epithelium. For each gene G, artificial expression level is:

$$ A G= k G*\left(\left(1- pE\hbox{'}\right)* mMG+ pE\hbox{'}*\left( pP\hbox{'}* mEG+\left(1- pP\hbox{'}\right)* mKG\right)\right) $$

First, to measure the impact of epithelium proportion on the position of the sample on the first axis of the PCA, we sampled artificial teeth corresponding to the expression levels ED14.5 with a range of plausible initial conditions (pE). We made several simulations, with plausible initial K compositions (pP in the range of 5–20% of the total epithelium) and with a range of coherent epithelium proportions (pE' representing 45–60% of the total tooth). From these simulations, we then built artificial samples in which the proportion of epithelium is increased and projected the obtained artificial samples on the PCA. On average, 15.7% of the PCA1 distance between ED14.5 and ED15.0 is covered when pE' increases by 30% (maximum = 28%, obtained when EK represents 20% of the epithelium).

Second, to measure the impact of non-enamel knot proportion (pP) on the position of the sample on the second axis of the PCA, we sampled an artificial tooth corresponding to a ED14.5 lower molar transcriptome and, from this, made artificial samples in which the proportion pP' of epithelium decreases. Starting from 55% non-EK epithelium and decreasing to 40% or 50% covers 23–35% of the real distance between upper and lower tooth on PCA2 at ED14.5.

### Estimating tissue proportions from RNA-seq data: deconvolutions

Performing gene expression deconvolution is estimating cell type proportions (and/or cell-specific gene expression signatures) from global expression data in heterogeneous samples. Here, we used the R package CellMix (CellMix_1.6.2 [97]) to estimate the proportions of the three tissue compartments in our tooth germ transcriptomes. CellMix implements, in particular, the method Digital Sorting Algorithm (*DSA*) proposed by Zhong et al. [98] which performs complete gene expression deconvolution using a set of marker genes only.

We needed therefore to define three sets of markers, as specific as possible for each tissue compartment. We first used markers obtained from bite-it database (used in Fig. 3b): 11 for EK, 12 for epithelium, and 14 for mesenchyme. In the case of the study of maturation, we further restricted EK markers to six genes with a large pattern of expression (used in Fig. 6g). The results were confirmed with markers obtained from microarray data ([39]; used in Additional file 1: Figure S3): 18 EK (defined such as expression level in ek > 1.2× expression level in mesenchyme and level in ek > 1.2× level in epithelium), nine for epithelium (such as epithelium > 1.5× mesenchyme and epithelium > 1.2× ek), and 27 mesenchyme (such as mesenchyme > 1.5× epithelium and mesenchyme > 1.2× ek). The thresholds used (1.5× and 1.2×) were set up to guarantee enough markers relatively equilibrated among the three tissues and yet that are specific enough for the tissue under study. Of note, results were qualitatively unchanged when the thresholds are modified (within a range 1.2–1.5 for both). To get an idea of the sensitivity of the results to the choice of the set of markers, for each analysis, we computed 500 bootstrapped values by randomly resampling half of the total number of markers (keeping only samples such as there are more than five markers per compartment) and recomputing deconvolutions on these 500 random subsets. The confidence intervals of these resamplings are indicated in the barplots (Figs. 3b and 6g, Additional file 1: Figure S3).

### Morphological determination of the volume of tissue components

The mouse embryos and fetuses were harvested during ED14.5–17.5, weighted and fixed in Bouin-Hollande fluid. At each stage, one to two representative specimens exhibiting median body weight were selected. Their heads were routinely embedded with paraffin and series of 10 um thick frontal histological sections were prepared and stained by hematoxylin & eosin (H&E). On each section, the surfaces of dental epithelium and dental mesenchyme were measured for the fours molars. From these data, total volumes of the two compartments were calculated.

### Measuring heterochrony on genes with a bell-shaped profile

To estimate the gene-specific heterochrony offset, we computed the cubic splines of both expression curves (upper and lower): up(t) and lo(t). We model the offset h between up and lo as, for all times t:

$$ up(t)= l o\left( t+ h\right)+\epsilon $$

where

$$ \epsilon \sim N\left(0,1\right). $$

To compute the maximum likelihood estimation of h, we considered 100 equidistant time points in the whole interval, t_1_,…, t_100_; for any tested value of h, we kept all time points t'_i_ such that t'_i+h_ is also in the interval, and computed the average squared difference:

$$ \frac{1}{numberoft{\hbox{'}}_i}{\displaystyle \sum }{\left( up\left( t{\hbox{'}}_i\right)- lo\left( t{\hbox{'}}_{i+ h}\right)\right)}^2 $$

We define the heterochrony for a gene as the value of h which minimizes this measure.

### Method based on hidden Markov Models to model upper/lower differences

We designed a method, based on hidden Markov Models, to track differences in time profile, in a way which explicitly models the temporal series. First, we looked for a description in two classes of the overall distribution of the absolute value of upper/lower ratios, taking into account the temporal constraint. To do this, we modeled this distribution as a mixture of two normal distributions, organized in a hidden Markov model. This makes seven free parameters to estimate, using eight time points from 2477 genes. These two distributions (hidden states) correspond to medium (ME) and high (HI) upper/lower difference in expression levels. Considering all time series independently with the classical forward algorithm, it is possible to compute the likelihood of the whole data and we optimized this likelihood to fit at best the transition probabilities between the states of the hidden Markov Model the whole data (2477 genes with a differential expression between upper and lower), using R library RHmm version 2.0.3 (https://github.com/msemon/ToothTranscriptomeAnalyses). Finally, from this optimized hidden Markov Model, we computed the a posteriori probabilities of transition between states for each gene, in each interval of time points in each time series. In an attempt to better fit the distribution of the ratios, we performed similar analyses, using a hidden Markov model with two hidden states, where each hidden state is now modeled by a mixture of Gaussian distributions. In Fig. 4b, for each type of transition and each interval of time points, we counted how many genes followed this transition with a probability above 0.8. We also obtained similar results using a hidden Markov Model with three hidden states.

### Models of cusp maturation

Within a mouse litter, embryos of the same weight are rigorously at the same developmental stage. Therefore, to estimate the number of cusp patterned for a given RNA-seq sample, we used an embryo harvested from the same litter and having the same weight. Following epithelium dissociation, we performed Fgf4 in situ hybridization to reveal the number and position of Fgf4 expressing EK. We built a simple model for quantifying cusp patterning and maturation in time, fueled with the data on cusp number and position mentioned above. For simplicity, we chose to model cusp proportions with a simple grid model of six (lower molar) or eight (upper molar) equally contributing units, corresponding to the cusps. Once patterned, the territory of each cusp expands at the same speed within a unit. (Fig. 6c). The expansion of the territory is simply coded by increments of one unit (from 0: no cusp patterned to 8: fully expanded cusp) per half day. The proportion of cusp tissue per tooth germ is then computed as the sum of the values assigned to each cusp, divided by the total volume for a fully patterned tooth (total number of cusps × maximal value = 8). In this model, the cusps are still being incremented at the last time point. Alternative models were built by incrementing the cusp proportions over y steps only after the cusp patterning (the model with five steps, corresponding to 2.5 days, has the best fit). The fit of these models was computed using generalized linear models (glm in R), which is modeling the proportion of cusp tissue over the total molar tooth germ.

### Estimating heterochrony in the different tissue compartments

Using microarray data [39], we selected three sets of markers corresponding to each tissue compartment, that contain a similar number of genes. We obtained 336 markers relatively specific to epithelial cells, such that the ratio epithelium/mesenchyme is among the top 5% and the ratio epithelium/EK is in the top 20%. Similarly, we selected 421 EK markers (EK/epithelium in the top 5% and EK/mesenchyme in the top 5%) and 566 mesenchyme markers (mesenchyme/epithelium in the top 5% and mesenchyme/EK in top 5%).

By drawing PCAs based on these subsets of genes that are relatively specific from each tissue compartment, we obtained a map of the samples seen by this tissue. This permits to evaluate whether each tissue is carrying the time component and the heterochrony seen in the whole dataset. Furthermore, as explained briefly in Additional file 1: Figure S6a, the normalization of expression data only on these genes removes part of the influence of tissue composition.

## Additional files

Additional file 1: Supplementary figures S1-S7 and supplementary text. **Figure S1.** First axis of the lower-specific and upper-specific PCAs orders samples with time. **Figure S2.** Gene ontology analysis of the genes robustly assigned to one of the ten clusters obtained for lower and upper molar. **Figure S3.** Mesenchyme proportion estimated using deconvolutions with markers based on microarray data. **Figure S4.** Mesenchyme proportion are always larger in upper molar as measured from 3D reconstructions of dissected tooth germs. **Figure S5.** Models of cusp patterning and expansion in the lower and upper molars. **Figure S6.** Heterochrony signals are visible in the transcriptomes of each tissue compartment. **Figure S7.** In a limb development dataset, PCA1 coordinates correlate with proportions of chondrocytes estimated by deconvolution (see supplementary text). **Supplementary text.** Time signal and heterochrony in limb development can be interpreted alongside with estimation of the proportion of differentiated cell types. (PDF 3938 kb)
Additional file 2: Table S1. Genes differentially expressed in ED14.5–18.0 molars. An excel file with data for genes differentially expressed under various conditions in our study. Sheet 1: content and legend. Sheet 2: data for genes which are significantly differentially expressed in at least one out of three comparisons done with DEseq2 (eight stages taken as replicates; eight stages taken as replicates, taking time into account as a quantitative variable; one out of the seven pairs of consecutive stages taken as replicates): log ratio, *P* value, mean basemean. (XLSX 422 kb)
