# Supplementary material for: Transcriptomic signatures shaped by cell proportions shed light on comparative developmental biology
Source: Genome Biol. 2017 Feb 15;18:29. doi: 10.1186/s13059-017-1157-7 (PMC5312534; doi:10.1186/s13059-017-1157-7)
Supplement: Additional file 1: — Supplementary figures S1-S7 and supplementary text. Figure S1. First axis of the lower-specific and upper-specific PCAs orders samples with time. Figure S2. Gene ontology analysis of the genes robustly assigned to one of the ten clusters obtained for lower and upper molar. Figure S3. Mesenchyme proportion estimated using deconvolutions with markers based on microarray data. Figure S4. Mesenchyme proportion are always larger in upper molar as measured from 3D reconstructions of dissected tooth germs. Figure S5. Models of cusp patterning and expansion in the lower and upper molars. Figure S6. Heterochrony signals are visible in the transcriptomes of each tissue compartment. Figure S7. In a limb development dataset, PCA1 coordinates correlate with proportions of chondrocytes estimated by deconvolution (see supplementary text). Supplementary text. Time signal and heterochrony in limb development can be interpreted alongside with estimation of the proportion of differentiated cell types. (PDF 3938 kb) [file 13059_2017_1157_MOESM1_ESM.pdf]

Additional file 1: Supplementary figures and text

Content

Sup Fig 1: First axis of the lower and upper-specific PCAs orders samples with time------- p.2

Sup Fig 2: Gene ontology analysis of the genes robustly assigned to one of the 10 clusters obtained for lower and upper molar-- p. 3

Sup Fig3: Mesenchyme proportion estimated using deconvolutions with markers based on microarray data--- p. 4

Sup Fig 4: Mesenchyme proportion are always larger in upper molar as measured from 3D reconstructions of dissected tooth germs -- p.5

Sup Fig 5: Models of cusp patterning and expansion, in the lower and upper molar ------ -p. 7

Sup Fig 6: Heterochrony signals are visible in the transcriptomes of each tissue compartment--- p. 10

Sup Fig 7: PCA1 coordinates of the different timepoints in the limb dataset by Taher et al. 2011 correlate with proportions of chondrocytes estimated by deconvolution. ------------- p. 12

Supplementary text: time signal and heterochrony in limb development can be interpreted alongside with estimation of the proportion of differentiated cell types ----------------------- p. 12

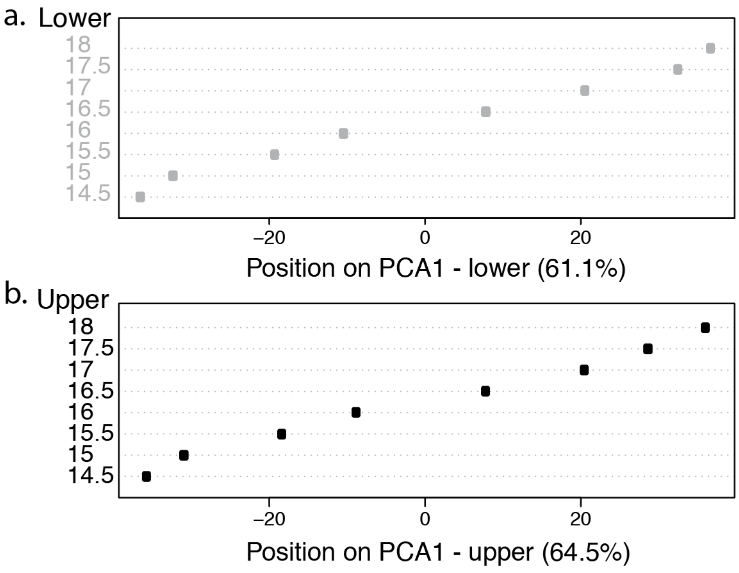

SUP Fig 1. First axis of the lower and upper-specific PCAs orders samples with time. (a) The location of lower molar germs on the first axis of a PCA drawn specifically with the 8 lower samples. **(b)** The location of upper molar germs on the first axis of a PCA drawn specifically with the 8 upper samples.

SUP Fig 2. Gene ontology analysis of the genes robustly assigned to one of the 10 clusters obtained for lower and upper molar The columns represent the ten main temporal profiles of expression in the lower (**a.** top) and upper (**b.** bottom) tooth, as described Figure 3. Cluster numbering is random (same as in Figure 3), and do not coincide between upper and lower tooth. The number of genes is indicated for each cluster, and the corresponding profile is in represented in bold. Lists of genes assigned to a each cluster were submitted to GO analysis using GOstats. Enrichment for a list of relevant GO terms (grouped by 8 broad functional category, see colors on the left side) are represented. The width of each bar is proportional to the size of the GO term (in number of genes). The length of each bar represents the enrichment (measured by an odd ratio), orange bars corresponding to GO Terms which are significantly enriched in the analysis (adjusted pvalue <0.1), and purple bars corresponding to GO Terms which are not significantly enriched. n.d means that no genes assigned to this GO Term belong to the cluster.

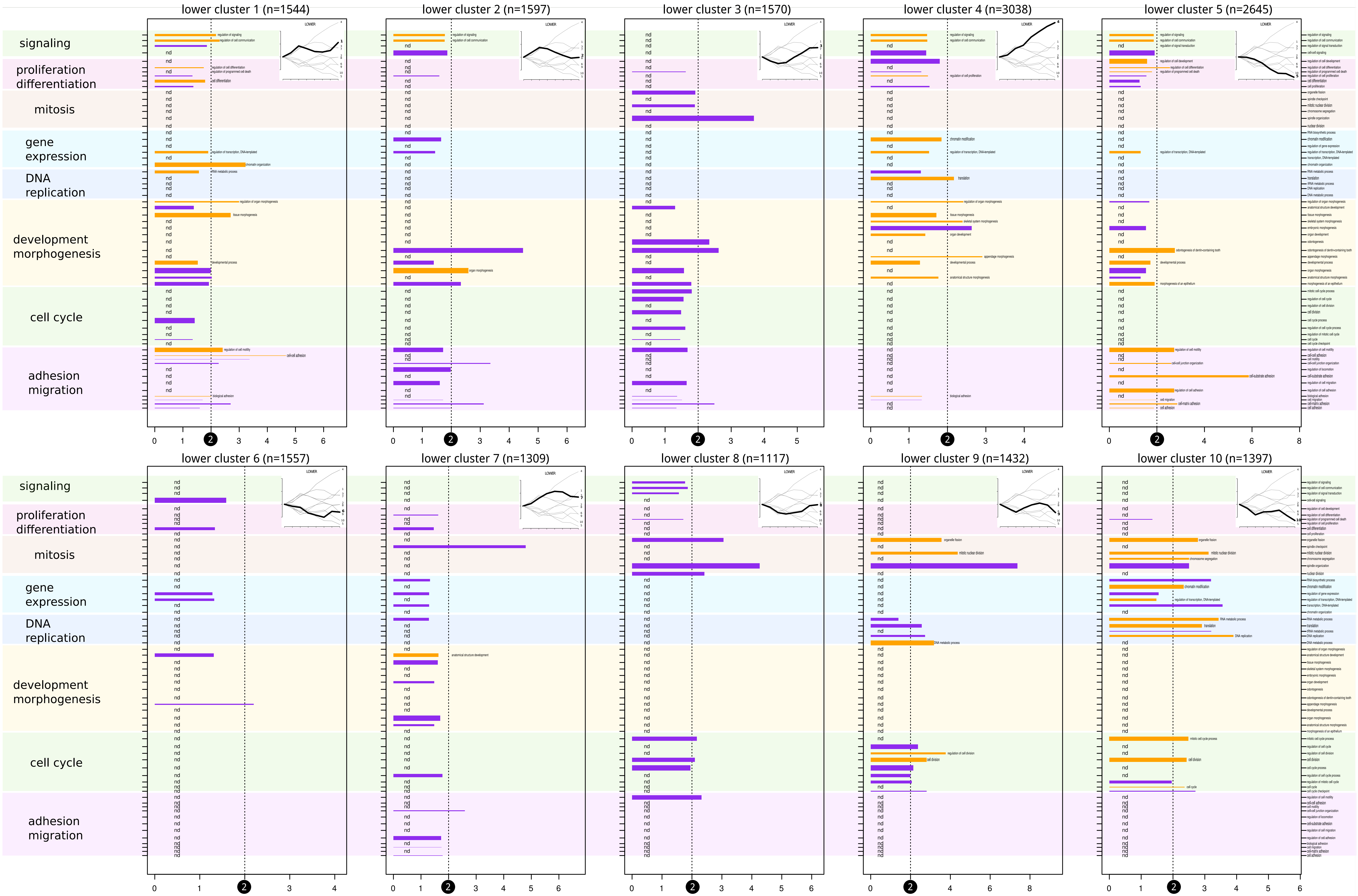

lower cluster 1 (n=1544)
lower cluster 2 (n=1597)
lower cluster 3 (n=1570)
lower cluster 4 (n=3038)
lower cluster 5 (n=2645)
lower cluster 6 (n=1557)
lower cluster 7 (n=1309)
lower cluster 8 (n=1117)
lower cluster 9 (n=1432)
lower cluster 10 (n=1397)
signaling
proliferation differentiation
mitosis
gene expression
DNA replication
development morphogenesis
cell cycle
adhesion migration
LOWER
nd

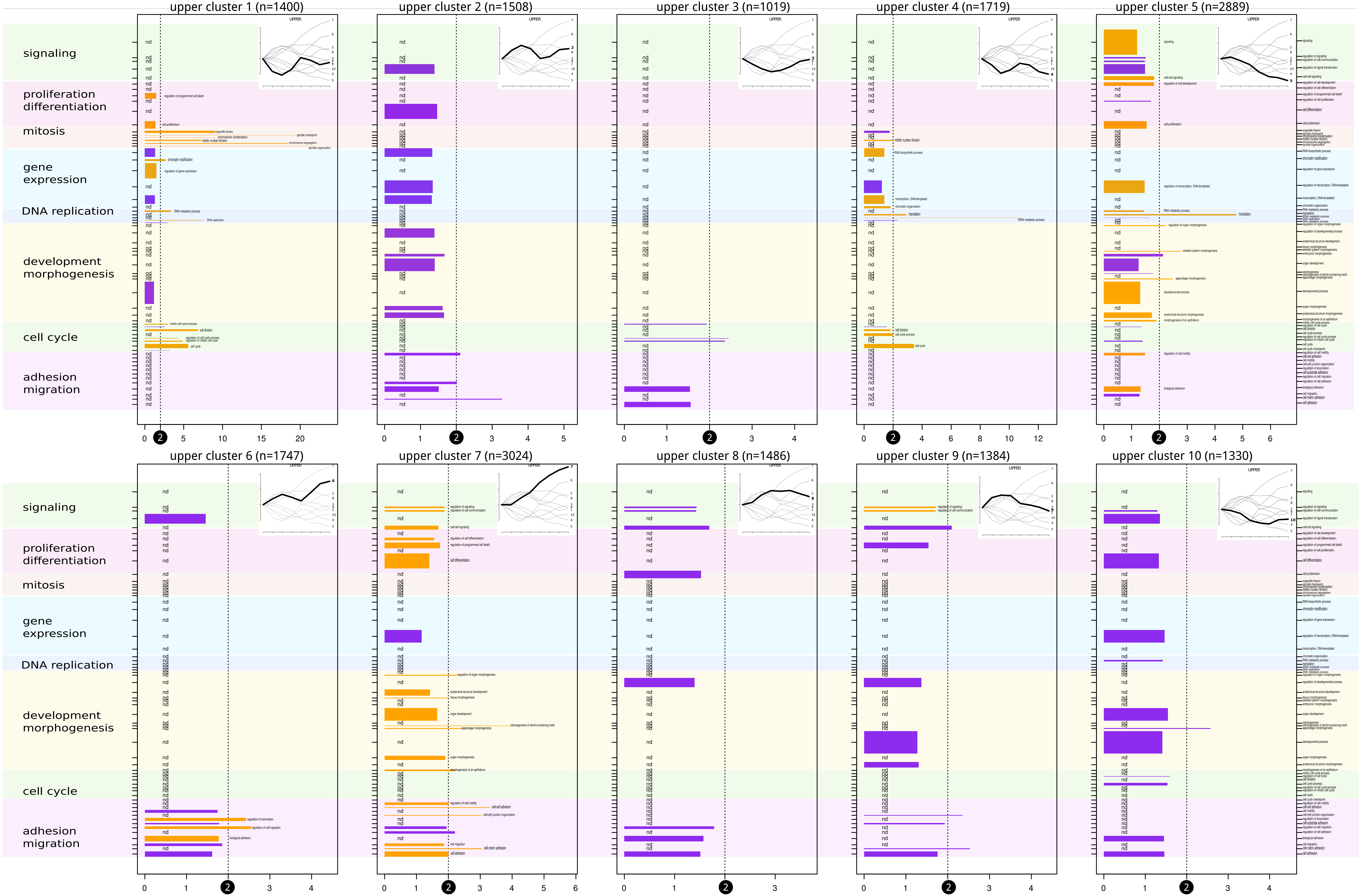
upper cluster 1 (n=1400)
upper cluster 2 (n=1508)
upper cluster 3 (n=1019)
upper cluster 4 (n=1719)
upper cluster 5 (n=2889)
upper cluster 6 (n=1747)
upper cluster 7 (n=3024)
upper cluster 8 (n=1486)
upper cluster 9 (n=1384)
upper cluster 10 (n=1330)
signaling
proliferation differentiation
mitosis
gene expression
DNA replication
development morphogenesis
cell cycle
adhesion migration
UPPER
nd

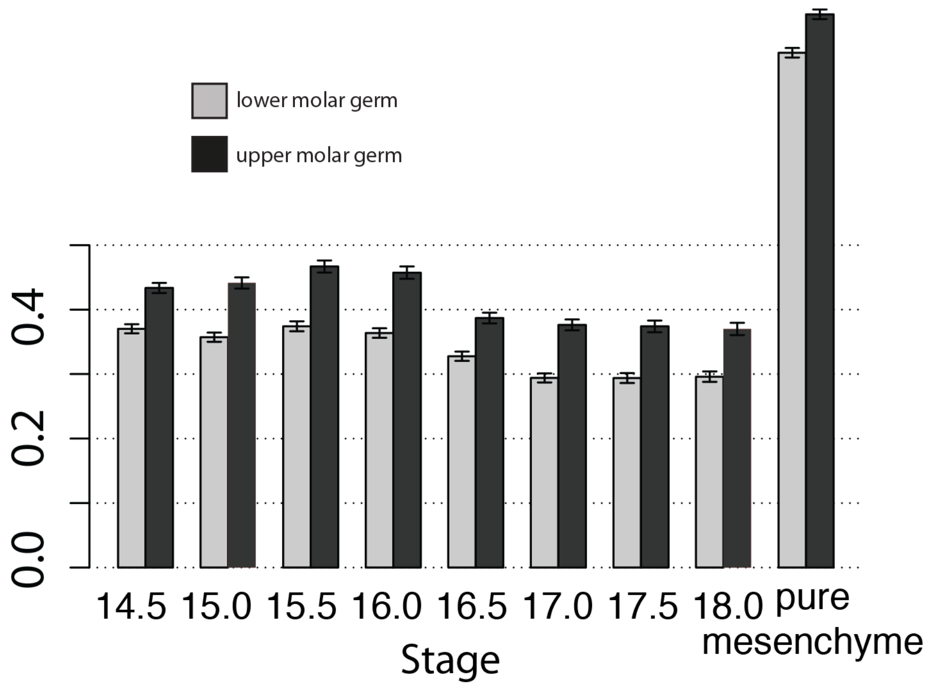

SUP Fig 3. Mesenchyme proportion estimated using deconvolutions with markers based on microarray data.

Markers were obtained from microarray data ([36]; 18 for EK, 9 for epithelium and 27 for mesenchyme). Confidence intervals were extracted by resampling (500 bootstraps). RNA-seq samples for tooth mesenchyme [42] were used as positive controls.

a. A series of confocal sections of a dissected ED15.0 lower molar germ stained with pan-cytokeratin antibody

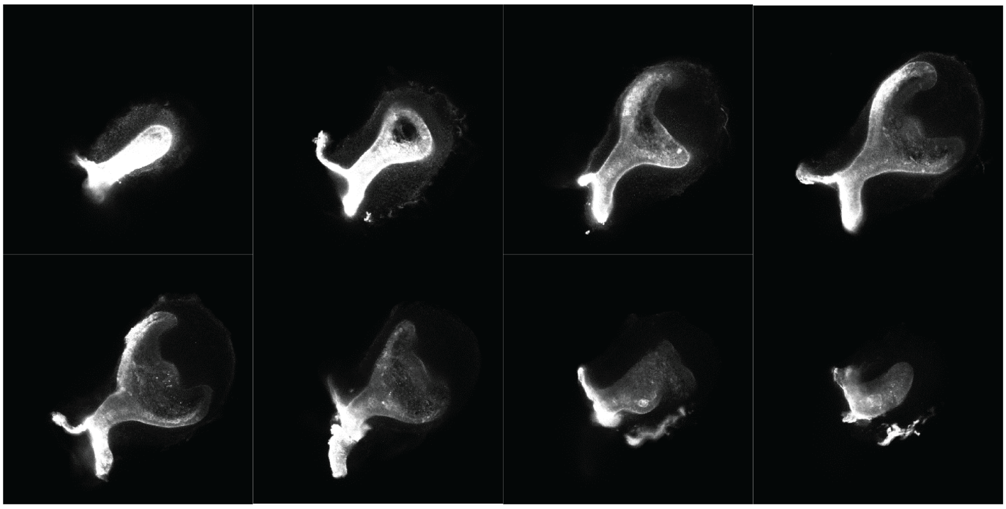

b.Epithelial component and total whole germ 3D reconstructions from the sections shown in a.

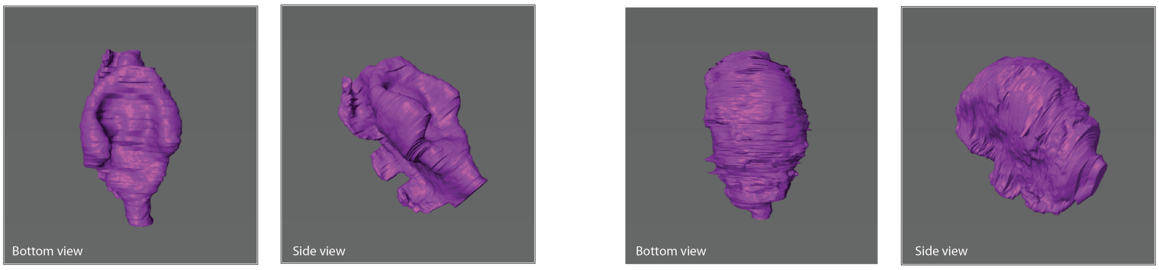

3D-reconstructed epithelial component

3D-reconstructed total molar germ

c. Measure of mesenchyme proportion from 5 pairs of 3D- reconstructed lower-upper molar germs

		measured volumes		% of total volume		
		Epithelium volume	Total volume	Epithelium	mesenchyme	% Vmes(upper)-%Vmes(lower)
ED14.5	lower	5030708	14011360	35,9	64,1	
	upper	6833440	23084740	29,6	70,4	6,3
ED14.5	lower	5984882	20390236	29,4	70,6	
	upper	7109809	26925336	26,4	73,6	3,0
ED15.0	lower	5004660	12344049	40,5	59,5	
	upper	5154652	13540549	38,1	61,9	2,5
ED15.0	lower	9356574	32496996	28,8	71,2	
	upper	10398992	40934108	25,4	74,6	3,4
ED15.5	lower	14238123	36044588	39,5	60,5	
	upper	13448003	37602856	35,8	64,2	3,7

SUP Fig 4. Mesenchyme proportion are always larger in upper molar as measured from 3D reconstructions of dissected tooth germs.

(a) A series of confocal sections of a dissected ED15.0 lower molar germ stained with pan-cytokeratin antibody

(b) Epithelial component and total whole germ 3D reconstructions from the sections shown in (a).

(c) Table showing measures of mesenchyme proportion from 5 pairs of 3D- reconstructed lower-upper molar germs.

Supplementary material for this figure: Tooth germs dissected from littermate embryos of RNA-seq samples (same weight) were fixed overnight in PFA4% and dehydrated through a methanol series. In toto immunolocalisation protocol was adapted from *Ahnfelt-Ronne et al. J Hist Cytochem 2007 55(9):925-30*. Following incubation in H202 5%, DMSO 10% in methanol for 4 hours, they were rehydrated, blocked and incubated successively with a pan-cytokeratin antibody (overnight,1/200, Novus Biologicals) and a Dylight 549 conjugated Donkey Anti-rabbit antibody (overnight 1/200, Jackson immunoresearch) and finally with Hoechst (overnight , 50ug/ml). Following dehydration, they were clarified and mounted in BABB as described in *Yokomizo and Dzierzak Development 2010, 137 :3651-61*. They were imaged with a Zeiss LSM710 confocal microscope at the PLATIM (Lyon, France). The outline of the epithelium labeled by the pan-cytokeratin antibody and the outline of the tooth germ labeled with hoechst were delineated manually in the AMIRA software. Total epithelium and total tooth germ were reconstructed in 3D and their volume measured in the AMIRA software as well.

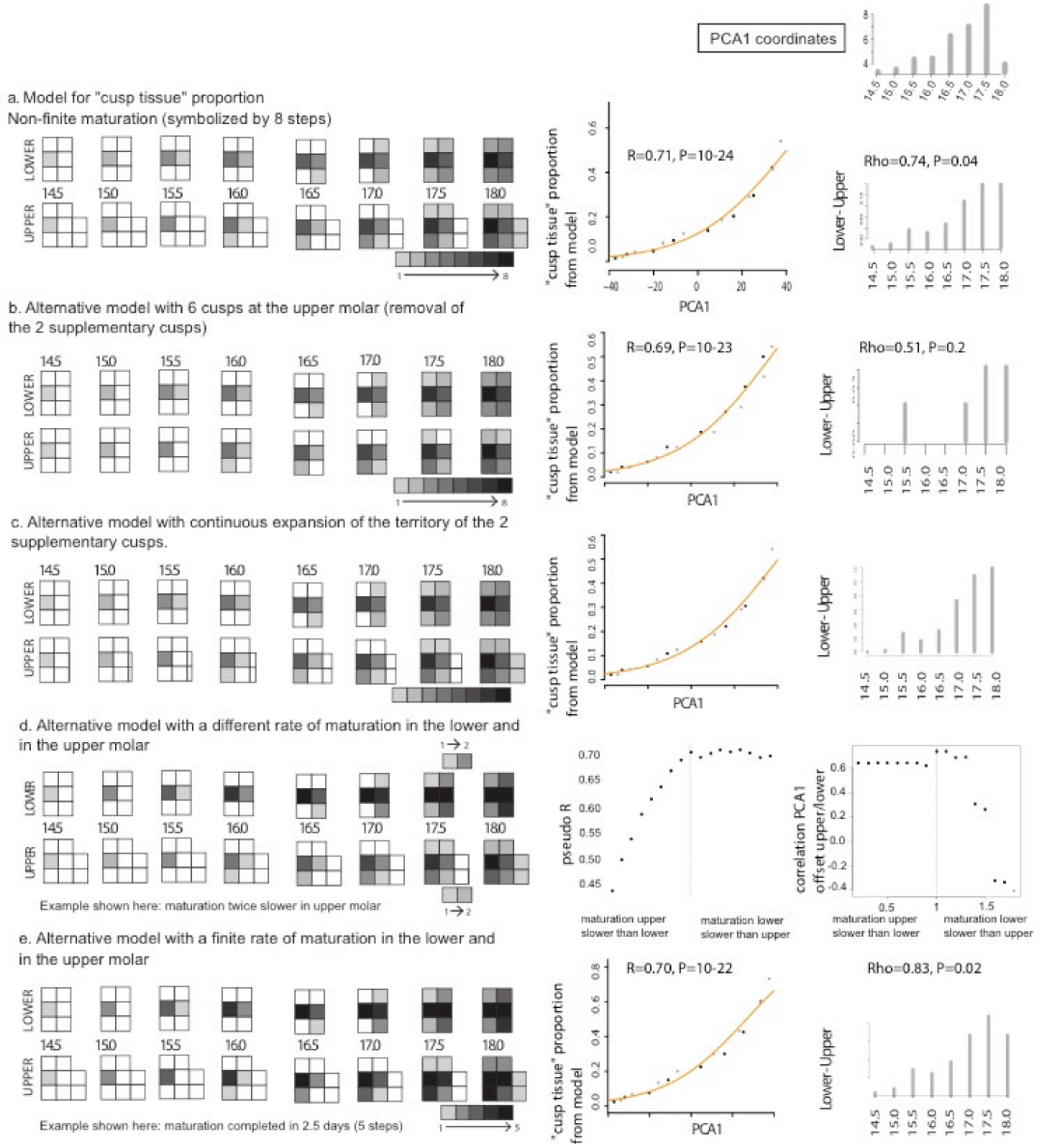

SUP Fig 5 Models of cusp patterning and expansion, in the lower and upper molar.

Model of cusp expansion in the lower and upper molars were build using in situ data on the timing of cusp patterning. Once patterned, the territory of each cusp expands at the same speed. In the schematic representation, the average shade of grey of each tooth, at each developmental stage, gives a visual impression on the average degree of the expansion of the cusp territory, from unpatterned cusp (white) to just-patterned cusp (light grey) and fully

expanded cusp (black). We computed cusp expansion in each tooth based on this model, 0 corresponding to no cusp patterned (all white), and 1 to a complete expansion (all black), which we will later refer to as “cusp tissue” proportion from model.

(a) Left : Model applied to the upper and lower tooth, following the sequence of cusp addition that ranges from 1 to 8 cusps in upper molar, and from 1 to 6 cusps in lower molar (see Figure 5e)**;** The time for complete expansion is set to 8 steps, so that the first cusp to pattern (at stage 14.5) is fully expanded at the last stage of the series (at stage ED 18.0). Middle : y axis represents the estimated “cusp tissue” proportions from the model, x axis represents the position of the samples on the first PCA axis. P-value associated to a binomial regression = 10-24, McFadden's pseudo R squared = 0.71. Right : Offset of cusp proportion as estimated from the model (corresponding to Lower-Upper), for each developmental stage. With respect to the overall fit to PCA1 (middle column), none of the three alternative models (**b-d**) perform better than this main model (a), and they are all worse considering specifically upper/lower heterochrony. Legend for b-d follows (a).

(b) First alternative model, in which the two supplementary cusps are omitted. This implies that the formation of extra cusps does not translate in a change in cusp proportions. Middle : P = 10-23, pseudo R squared = 0.69.

(c) Second alternative model, in which upper tooth is progressively acquiring the extra space corresponding to the extra cusps. Middle: P = 10-22, pseudo R squared = 0.68.

(d) Third alternative model, in which the rate of maturation is different in the lower and upper molars. Right: The example shows a 50% slower rate in upper molar. Middle : P = 10-23, pseudo R squared = 0.69. Middle : We build 17 models by making the relative rates of maturation vary between upper and lower molars (from a ratio of upper/lower rates = 0.2 to upper/lower rates = 1.8, by steps of 0.1). We assessed each model through McFadden's pseudo R squared, which are represented in the middle panel. It is obvious that models with upper/lower ratio < 1 (that is, upper having a slower rate than lower) fit less well to the PCA. We also assessed each model through the correlation between the heterochrony estimated by the model and the heterochrony estimated by the PCA1 (Right). It is obvious that the

heterochrony is best recapitulated by models with equals rates between upper and lower molars, or by models with slower maturation in lower molar. From the middle and the right panel taken together, it can be deduced that the best model(s) have roughtly equals maturation rates in upper and lower molars.

(e) Finite cusp expansion : Model like in (a) but with reducing the time for each cusp full expansion. We reduced this time, from 8 steps (corresponding to the entire time serie, therefore to the infinite maturation model mentioned above) down to 2 steps. The pattern of heterochrony observed with the PCA is best recapitulated by a finite model which allows a duration of 2 days and a half for each cusp to expand (5 days, exemplified here). Note that the heterochrony (right) is slightly better than the one observed in (a), in that it correlates better with PCA1 coordinates.

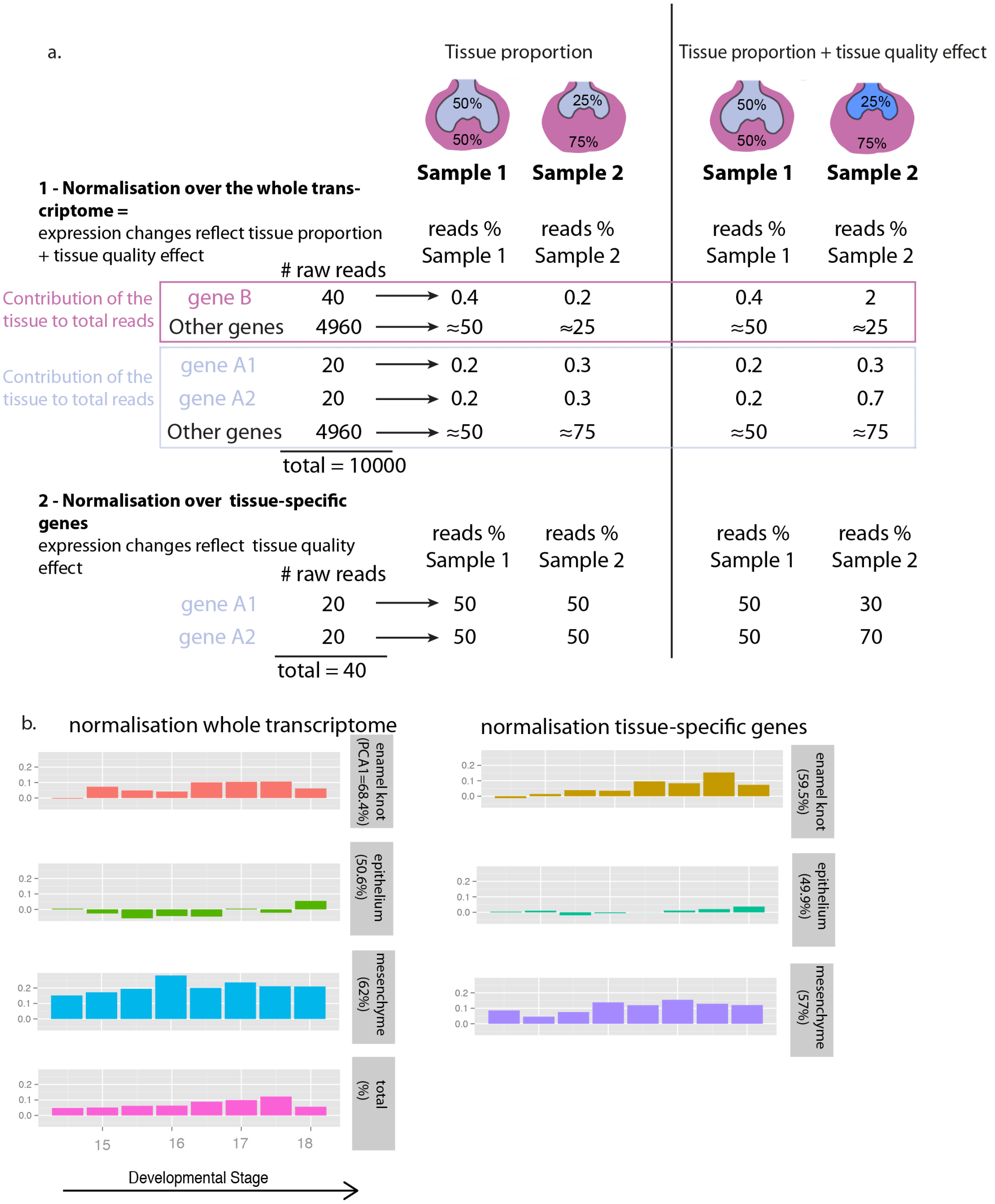

SUP Fig 6. Heterochrony signals are visible in the transcriptomes of each tissue compartment.

(a) Schematic explanation of the different effect measured when the normalization of RNAseq counts is performed on the whole transcriptome or over tissue specific genes only.

Two examples are taken.

Left: On the first example (tissue proportion, left) two samples are compared, for which cellular expression levels are the same (schematized here by the same pink and blue colors in both samples), but tissue proportions differ between samples (here, 50% epithelium in sample1, 25% epithelium in sample 2). Because of these differences in tissue proportions, the expression level of tissue-specific genes (like gene B, A1, A2 here) will be different between sample 1 and sample 2. The expression of gene A1, for instance, appears to be different when normalization is performed on the whole dataset, but is equal when normalization is done on tissue-specific genes.

Right: On the second example (right), both tissue proportions and cellular expression levels differ between stages (schematized here by a different blue color in the samples). The expression of gene A1, for instance, remains different when normalisation is done on tissue-specific genes.

(b) Heterochrony measured on PCA drawn using the whole dataset (total), and with 3 subsets of genes, each specific to one of 3 tissue compartments (336 epithelium markers, 421 EK markers and 566 mesenchyme markers). Normalization was done on the whole transcriptome (left) or on each set of markers (right, normalization on tissue-specific genes). For each dataset, the first axis ordered the samples with their developmental advancement. The height of each bar represents the heterochrony measured on the first PCA axis in each case (that is, coordinate of lower sample on PCA1 – coordinate of upper sample on PCA1, for each time point).

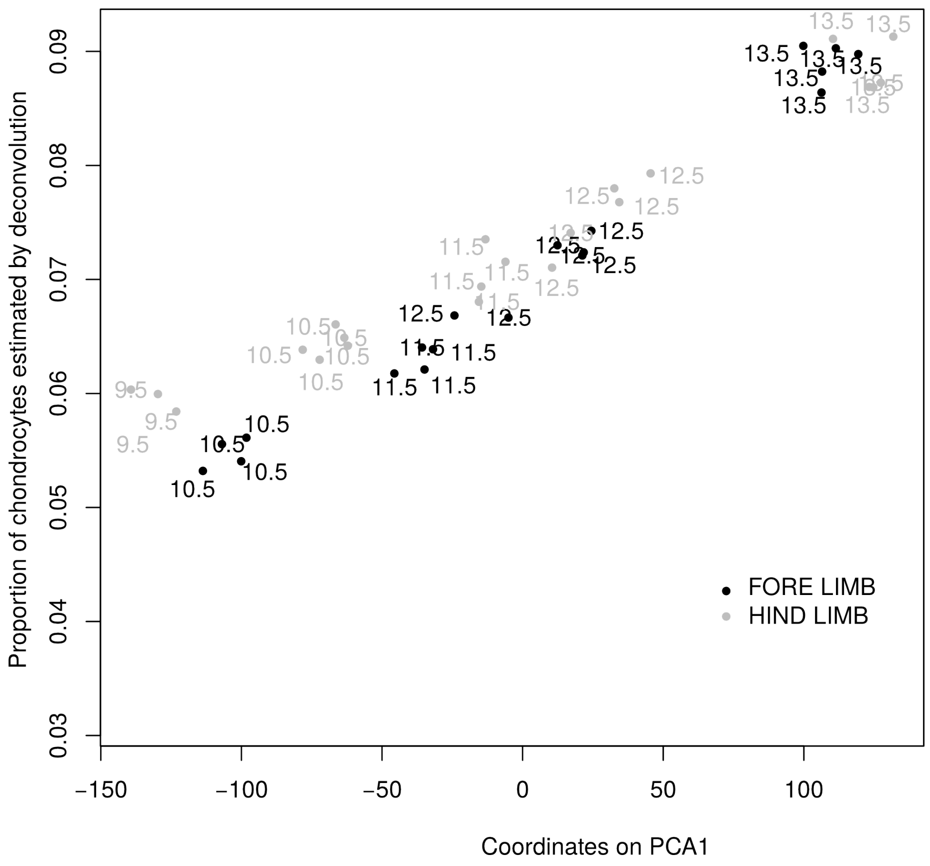

Fig S7: PCA1 coordinates of the different timepoints in the limb dataset by Taher et al. 2011 correlate with proportions of chondrocytes estimated by deconvolution. Timepoints are taken at 9.5 (forelimb), 10.5, 11.5, 12.5 and 13.5 (fore and hindlimb) days of gestation (see supplementary text below for details).

Supplementary Text

To see whether our findings can be generalized to other serial organs, we re-analysed the most comprehensive published transcriptome dataset on fore/hind limb development, with expression profiled by microarrays in many replicates for 20737 mouse genes at 4/5 stages of limb development (Taher et al. 2011). The PCA analysis of this dataset is clearly revealing a temporal signal on the first axis (PCA1 = 31.5% of the global variation; Fig 7C). We tested the significance of this temporal signal by a linear model (p-value < 10^{-16}). The addition of

the type of organ further improves the fit of the model (p-value = 2*10-05). This is demonstrating that heterochrony between fore and hind limb is visible from the transcriptomes, forelimbs being advanced compared to hind limbs. This is a well-known characteristics of mouse limb development.

An obvious feature of the advancement of limb differentiation is that it should correlate with the advancement of chondrogenesis. From a published transcriptome analysis of chondrogenesis, we obtained 50 marker genes corroborated by the literature (Additional file 3 in [102]). PCA analysis based on these 50 marker genes show a temporal axis which is twice higher than the one obtained on the whole transcriptome (PCA1: 59.3% compared to 31.5% for the whole transcriptome).

Deconvolutions from these marker genes clearly show an increase of the proportion of chondrocytes during differentiation in the whole limb transcriptomes (same method as for the tooth dataset; average proportions from 100 random sampling of half of the chondrocyte markers; Fig 7C, anova test p-value <10-16). The proportion of chondrocytes estimated by deconvolutions and the coordinates on PCA1 are extremely well correlated (R=93%, p-value <10-16; Fig S7 below). The relative proportion is always higher in forelimbs compared to hindlimbs at comparable stages of development (Fig 7C, anova test p-value = 0.0014). So in conclusion, with an independent model of serial organs and with another type of data, we recover a strong time-related signal, and heterochronies in the transcriptomes. Both can be interpreted alongside with estimation of the proportion of differentiated cell types.
